# Capture of CO_2_ by Melamine Derivatives: A DFT Study Combining the Relative Energy Gradient Method with an Interaction Energy Partitioning Scheme

**DOI:** 10.1021/acs.jpca.3c08412

**Published:** 2024-02-13

**Authors:** Maxime Ferrer, Ibon Alkorta, Jose Elguero, Josep M. Oliva-Enrich

**Affiliations:** † Instituto de Química Médica (CSIC), Juan de la Cierva, 3, E-28006 Madrid, Spain; ‡ PhD Program in Theoretical Chemistry and Computational Modeling, Doctoral School, Universidad Autónoma de Madrid, 28049 Madrid, Spain; § Instituto de Química-Física “Blas-Cabrera” (CSIC), Serrano, 119, E-28006 Madrid, Spain

## Abstract

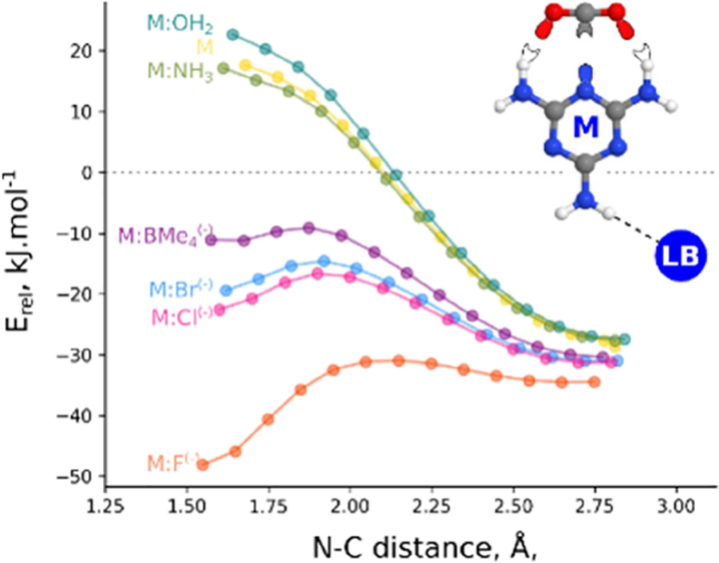

A theoretical study of the interaction between melamine and CO_2_ was carried out using density functional theory (DFT) with the B3LYP-D3(BJ)/aug-cc-pVTZ level of theory. The presence of anions interacting with melamine transforms the weakly bonded tetrel complexes into adducts. Thus, melamine acts as an FLP (frustrated Lewis pair) with acid groups (NHs as hydrogen bond donors) and a base group (N of the triazine ring). The application of the relative energy gradient formalism (REG) along the reaction coordinate has demonstrated that the ability of the melamine-anion systems to capture CO_2_ is linked to its capacity to polarize the CO_2_ molecule. These results have been confirmed by placing the melamine:CO_2_ complex in a uniform electric field with different strengths.

## Introduction

In 2022, the atmospheric CO_2_ concentration reached an alarming new record with an average concentration of 417 ppm over the year.^1−3^ Adding to this record the direct link existing between climate change, global warming, greenhouse effect and atmospheric CO_2_ concentration,^4−6^ the future of planet Earth gets more and more dystopic. For that reason, the scientific community is very concerned and many efforts are directed toward the research for new techniques and methods to reduce the previously mentioned concentration. So far, the main existing technologies can be divided into three categories: absorption, adsorption and membrane technologies.^7^ The most used in industry is the absorption of CO_2_ by amine solution, in general a solution of monoethanolamine (MEA).^8−11^ However, the large energy needed for the regeneration of the solvent after capture makes this method far away from the ambitious but needed CO_2_ capture cost of $20/ton,^12^ proposed by several USA and European research programs.

**Figure 1:**
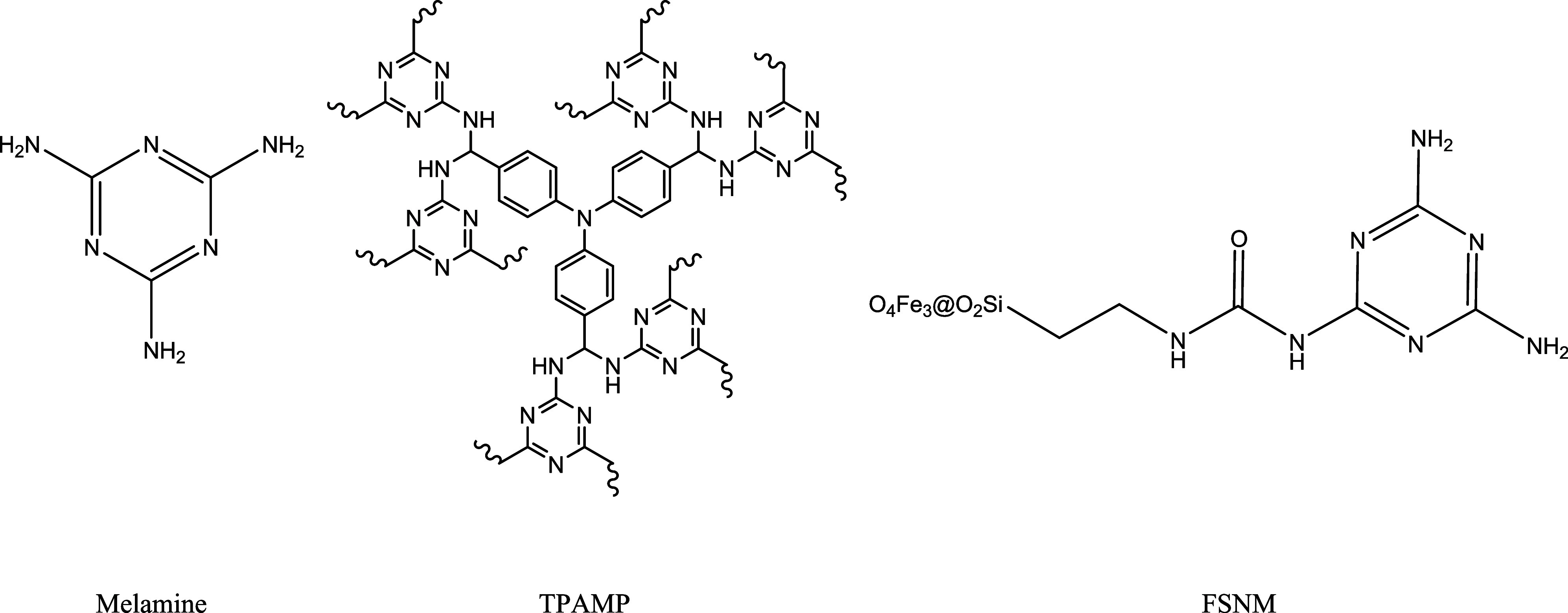
Melamine and two related systems are able to capture CO_2_.

A molecule that has gained more interest in recent years for CO_2_ capture is melamine.^13−17^ This nitrogen rich compound (Figure 1) can interact with CO_2_ through an acid–base interaction. It is an excellent building block as it is able to make external cross-linking with different functionalized monomers like ketones or carboxylic acids.^7^ The melamine also presents the ability of polymerizing, which makes it a perfect candidate for the preparation of resins capable of interacting preferentially with CO_2_. For example, by reacting with formaldehyde, it condensates into a mixture of methylol melamines and polymerizes to give the melamine-formaldehyde polymer.^18^ By some modifications of the last polymer, like the ones proposed by Pevida et al.,^13^ it is possible to improve the ability of the polymer to adsorb CO_2_. Tang et al.^19^ reported a melamine-based polymer able to catalyze the cycloaddition of CO_2_ to various epoxides (TPAMP Figure 1). In 2021, Guha et al.^20^ reported a similar reaction, but this time linking melamine to an iron oxide via carbamide linkage with one of the amine groups (FSNM Figure 1).

In the literature the adsorption of CO_2_ on melamine has been reported.^21,22^ Three interactions take place: one dative interaction between the lone pair of one of the pyridine type nitrogen atoms and the electron deficient carbon in CO_2_ (tetrel bond),^23−26^ and two hydrogen bonds between the CO_2_ oxygen atoms and the hydrogens of the NH_2_ groups. The authors report a N–C distance of around 2.90 Å and an O–H distance of around 2.30 Å and no formation of a covalent adduct. The adsorption of CO_2_ is based on a triple Lewis acid-Lewis base interaction. Even if melamine cannot be defined as a Frustrated Lewis Pair, its reactivity toward CO_2_ can be seen as a “FLP-type” reactivity.^27−29^ Thus, it can be thought that under certain conditions, the reactivity of melamine can be improved to obtain the typical FLP–CO_2_ adduct.

In this work, we look for conditions in which melamine can better act in absorption processes as compared to adsorption processes. Thus, the corresponding stationary points (minima and TSs) for the reaction between melamine derivatives and CO_2_ have been obtained by utilizing quantum-chemical computations. Based on these studies, it was observed that the presence of a charged moiety interacting with one of the amine groups favors the formation of a covalent adduct between melamine and CO_2_. Using the Relative Energy Gradient (REG) method^30−33^ coupled with the Non-Covalent Interactions Energy Decomposition Analysis,^34,35^ we have studied the adduct formation in the capture of CO_2_ by melamine derivatives.

## Computational Details

All the structures presented in this paper were optimized with the B3LYP functional^36,37^ and the aug-cc-pVTZ basis set^38,39^ including the D3 empirical dispersion with the Becke-Johnson damping, D3(BJ)^40^ to properly take into account the long-range electron correlation. Frequency calculations have been carried out at the same computational level to verify the nature of the stationary point found (no imaginary frequency for a minimum and one imaginary frequency for a transition state). These calculations have been carried out with the Gaussian16 software.^41^ The optimized geometries are gathered in the Supporting Information material.

The molecular electrostatic potential (MESP) of the different monomers studied were generated using B3LYP-D3(BJ)/aug-cc-pVTZ wave functions and the Multiwfn software.^42^ The MESP has been represented and analyzed on the 0.001 au electron density isosurface. Negative values of the MESP locate regions where a nonpolarizing positive charge will be attracted to. On the contrary, positive regions interact with negatively charged groups. In general, negative regions correspond to a local concentration of electrons, as, for example, lone pairs, while positive regions provide local depletion of electrons.

The Quantum Theory of Atoms in Molecules (QTAIM)^43,44^ has been used to perform a topological analysis of the electron density. In the present article, the AIMAll software^45^ was used to analyze the B3LYP-D3(BJ)/aug-cc-pVTZ wave function of the systems. By locating in space the points where the gradient of the electronic density vanishes, the molecule can be divided into atoms. It will be considered that two atoms interact if it is possible to localize a bond critical point (BCP) between them. A BCP is a particular point of the electron density that has nonzero Hessian eigenvalues, and it is a minimum along the bonding direction and a maximum in the two other perpendicular directions. Some properties at the BCP (density, Laplacian of the density, and electron density energies, among others) can help in characterizing the interaction under study.

A uniform electric field was applied to the melamine:CO_2_ model in the three spatial directions and pointing toward positive and negative values of each axis in order to check the importance of the polarization in analogy to previous studies that have used such methodology to study the proton transfer and H_2_ generation reactions.^46,47^

### Relative Energy Gradient Method

The Relative Energy Gradient (REG) method, developed by Thacker et al.,^30^ enables to understand a reaction using energy contributions. It was originally coupled with the Interacting Quantum Atoms (IQA) method,^48,49^ which decomposes the total electronic energy into atomic and diatomic energy contributions. REG highlights which of the numerous IQA components are the ones at the origin of an observed barrier in a reaction process. The main idea is to perturb a system along a given coordinate *s* (bond length, angle, RC, or others). For each perturbed configuration of the system, the total energy is calculated (*E*_tot_), and it is decomposed into energy contributions (*E*_*i*_). A set of functions depending on *s* (*E*_tot_(*s*); and *i* E_*i*_(*s*)) is then obtained. In fact, eq 1 is verified if the decomposition is exact.
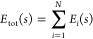
1

Then, the goal is to find how the total energy and the energy contributions respond to the perturbation and to identify the energy contributions that respond to the perturbation the same way the total energy does. This is done by doing linear regressions between each energy contribution and the total energy (eq 2). The slope of the linear regression, eq 3, is called the REG, and it is equivalent to the importance/weight of the contribution. The quality of the fitting is quantified by the Pearson correlation coefficient (eq 5).
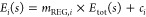
2
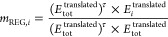
3
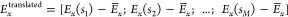
4
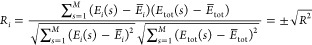
5

As already mentioned, the REG method was developed in order to find the IQA components controlling a given barrier. In this article, it is proposed to use the REG method with the energy decomposition scheme proposed by Mandado et al.^34^ In this case, the total relative energy (Δ*E*) is decomposed as deformation energy (*E*_def_) and interaction energy (*E*_int_) (eq 6). The interaction energy is also divided, based on the electron density, into electrostatic (*E*_elec_), Pauli (*E*_Pauli_), and polarization (*E*_pola_) contributions (eq 6). The dispersion calculated with the D3(BJ) method is included in the polarization term. The decompositions were run using EDA-NCI software.^34,35^
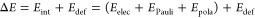
6

## Results and Discussion

This section has been divided into five subsections: (I) characteristics of the isolated melamine and CO_2_ and their interaction, (II) interaction of melamine with CO_2_ in the presence of neutral electron donors (bases) interacting with melamine, (III) interaction of melamine with CO_2_ in the presence of anions, (IV) application of the REG method on some systems to understand their ability to capture CO_2_, and (V) effect of an external electric field on the melamine-CO_2_ interaction.

### Melamine and CO_2_

**Figure 2:**
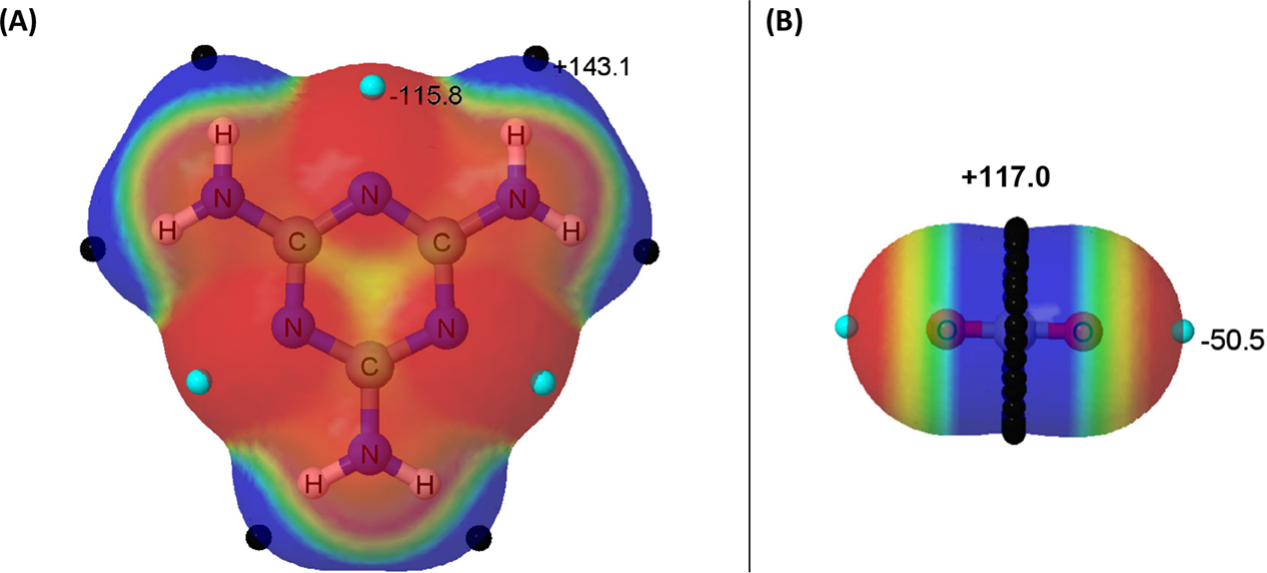
MEP of melamine (A) and carbon dioxide (B) on the 0.001 au electron density isosurface. The locations of the maxima and minima are indicated with black and light blue spheres. Their values is indicated in kJ mol^–1^.

The melamine molecule shows an effective *D*_3h_ symmetry lowered by the slightly pyramidal shape of the three amino groups. The alternated disposition of the three NH_2_ groups and three pyridine-type nitrogen atoms reminds us of the overlap of three guanidine groups forming a cycle. The molecular electrostatic potential of melamine on the 0.001 au electron density isosurface (MESP) is shown in Figure 2A, showing six positive regions associated with each NH bond and the three most negative electron-rich regions due to the lone pairs of the ring nitrogens. Thus, this molecule shows acid and basic centers in analogy to intramolecular FLP (it should be noted that some guanidines show activity as FLP when interacting with H_2_,^50^ CO_2_,^51^ and SO_2_^52^). The CO_2_ molecule shows a positive region around the carbon atom and two negative regions along the C–O bonds (Figure 2B).

The complex of melamine with CO_2_ shows three simultaneous interactions: a N···C tetrel bond (2.83 Å) and two symmetrical NH···O hydrogen bonds (2.24 Å), in good agreement with the complementarity shown by the MESP of the two molecules. Each of these interactions is associated with intermolecular bond critical points (BCPs) in the molecular graph of the complex with small values of ρ_BCP_ (0.013 and 0.012 au, respectively) and positive values of ∇^2^ρ_BCP_ (+0.041 and +0.049 au, respectively) as expected for weak noncovalent interactions.^53^ The calculated stabilization energy of the complex is −27.8 kJ mol^–1^ with respect to that of the isolated monomers.

**Figure 3:**
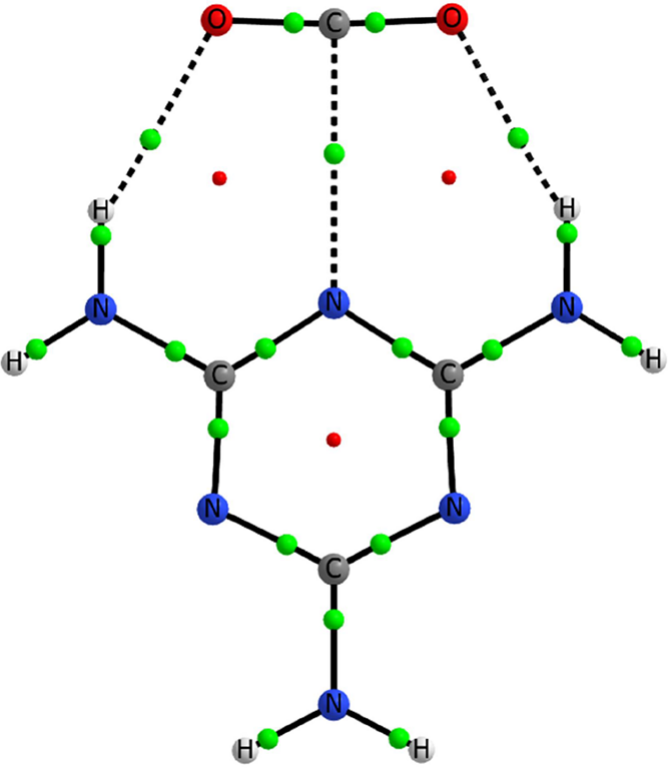
Molecular graph of the melamine:CO_2_ complex. The locations of the bond and ring critical points are indicated with medium green and small red spheres.

All efforts to obtain the corresponding adduct, i.e., the structure with a shorter N–C distance, spontaneously resulted in the formation of the complex shown in Figure 3. The approaching scan of the two molecules (Figure S1 of the Supporting Information material) reveals a continuous increase in energy as the two molecules approach, with a change in curvature around 2.0 Å, but no evidence of a minimum formation.

### Interaction of Melamine and CO_2_ in the Presence of Neutral Electron Donors

This section explores the electronic effects on the melamine molecule due to the formation of complexes with neutral electron donors and their influence on the interaction with CO_2_. Seven electron donors, NH_3_, NH_2_CH_3_, NH(CH_3_)_2_, N(CH_3_)_3_, OH_2_, O(CH_3_)_2_, and NCH have been selected.

**Figure 4:**
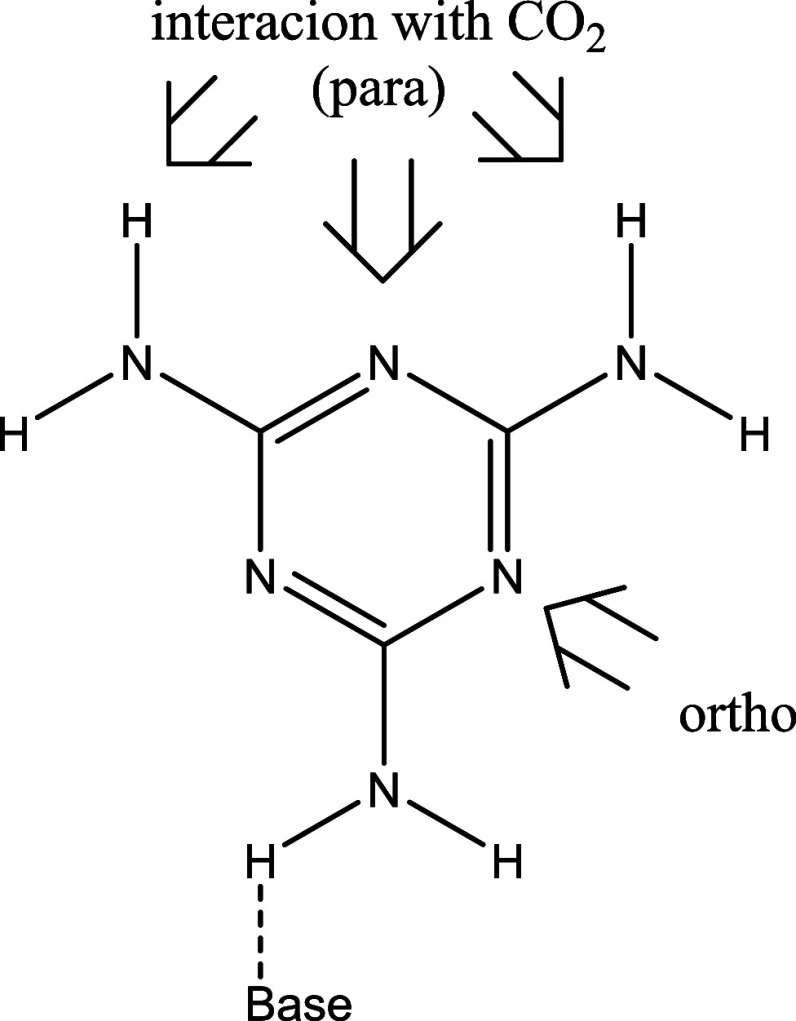
Schematic representation for the formation of melamine:base complexes and its interaction with CO_2_.

Two different approaches of the CO_2_ to the melamine:base have been considered, *para* and *ortho* (Figure 4). In all cases, the *para* approach provides more stable minima than the *ortho* one. Thus, in the main article, only the *para*-approach will be considered while the data corresponding to the *ortho* can be found in the SI.

The effect of the formation of the melamine:base complexes on the acid/basic properties have been evaluated using the calculated MESP minima and maxima since they are related to the basicity and acidity as measured by the proton affinity and the fluoride ion affinity, respectively.^54^ As shown in Table 1, the value of the MESP-minima associated with the nitrogen that will be involved in the interaction with CO_2_ increases (smaller absolute values) for the complexes with H_2_O, NH_3_, NH_2_CH_3_, and NH(CH_3_)_2_ and decreases (larger absolute values) for (CH_3_)_2_O, N(CH_3_)_3_ and HCN. It is interesting that the first group of bases acts as HB acceptors and donors while the second group only as acceptors. Thus, the influence of the HB donors weakens the basicity of the nitrogen while the HB acceptors increase it. In the presence of these two effects, the first is more important than the second one.

**Table 1:** MESP-Minima and MESP-Maxima Points (kJ mol^–1^) Associated to the N and H Atoms in the Melamine:Base Involved in the Interaction with CO_2_, and Binding Energies, *E*_b_, of the Melamine-Base:CO_2_ Complex (kJ mol^–1^)

M:base	*N*_*para*_	*H*_P1_, *H*_P2_	*E*_b_
M^a^	–115.8	+143.1, + 143.1	–27.8
M:H_2_O	–101.1	+155.2, + 155.7	–28.3
M:O(CH_3_)_2_	–120.2	+131.5, + 132.1	–27.7
M:NH_3_	–112.6	+140.7, + 138.4	–28.6
M:NH_2_CH_3_	–113.4	+139.7, + 137.6	–28.6
M:NH(CH_3_)_2_	–111.8	+141.5, + 139.2	–28.6
M:N(CH_3_)_3_	–120.0	+133.1, + 131.3	–28.7
M:NCH	–134.0	+126.0, + 127.1	–27.8

a M stands for melamine.

In all cases, a complex with CO_2_ is obtained with a stabilization energy between −27.7 and −28.7 kJ mol^–1^ (Table 1). Thus, the interaction between melamine and various electron donors minimally affects the stability of the resulting complexes. In agreement with the energetic results, the geometries of the new complexes obtained with CO_2_ are very similar to the melamine:CO_2_ geometries, with N···C tetrel interatomic distances ranging between 2.83 and 2.84 Å and H···O distances ranging between 2.24 and 2.25 Å. Likewise, the electron density properties at the three intermolecular BCPs are very similar to those found in the parent complex.

**Figure 5:**
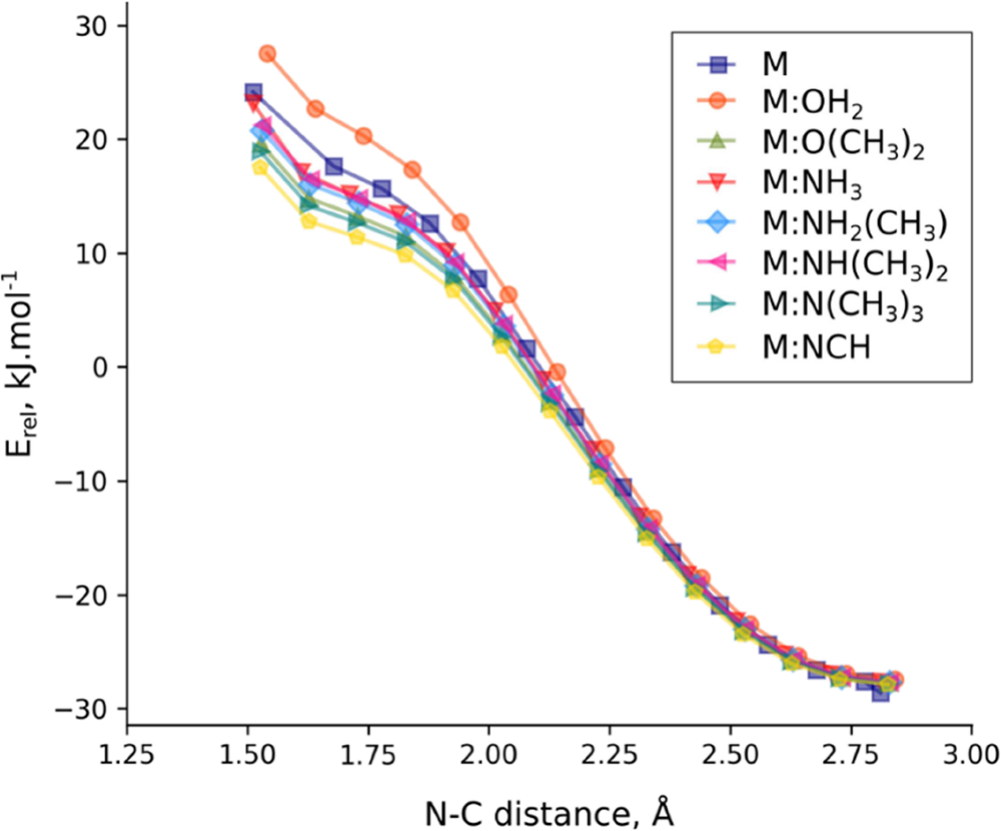
Relative energy vs N–C distance in the approach scan of the melamine-base:CO_2_ complexes.

All attempts to obtain adducts with CO_2_ revert spontaneously toward the complex previously described. In fact, the approaching scan of the melamine:base and CO_2_ systems (Figure 5) shows a profile with increasing energy as the two subsystems get closer with a change in the curvature in the N–C intermolecular distance around 2.0 Å as an indication that in the case of stronger bases, it will be possible to find the adduct.

### Interaction of Melamine and CO_2_ in the Presence of Anions

Based on the results from the previous section, one could, in principle, obtain melamine-CO_2_ adducts if the electronic properties of melamine are altered with stronger bases. Thus, we tried to capture CO_2_ with melamine-anion complexes. Six anions have been considered: B(CH_3_)_4_^(−)^, BF_4_^(−)^, CN^(−)^, Br^(−)^, Cl^(−)^, and F^(−)^. The anions have been selected based on their simplicity and covering a wide range of nucleophilicity: from low nucleophilic as B(CH_3_)_4_^(−)^ to very nucleophile ones, such as F^(−)^.

The complexes formed between melamine:anions and CO_2_ show binding energies between −29.5 and −33.6 kJ mol^–1^. These results indicate that the complexes are bound slightly more strongly compared to the ones with neutral bases. The N–C intermolecular distance (between 2.65 and 2.77 Å) in these complexes is slightly shorter than in the neutral systems, while the H···O distances are longer (between 2.25 and 2.26 Å).

**Figure 6:**
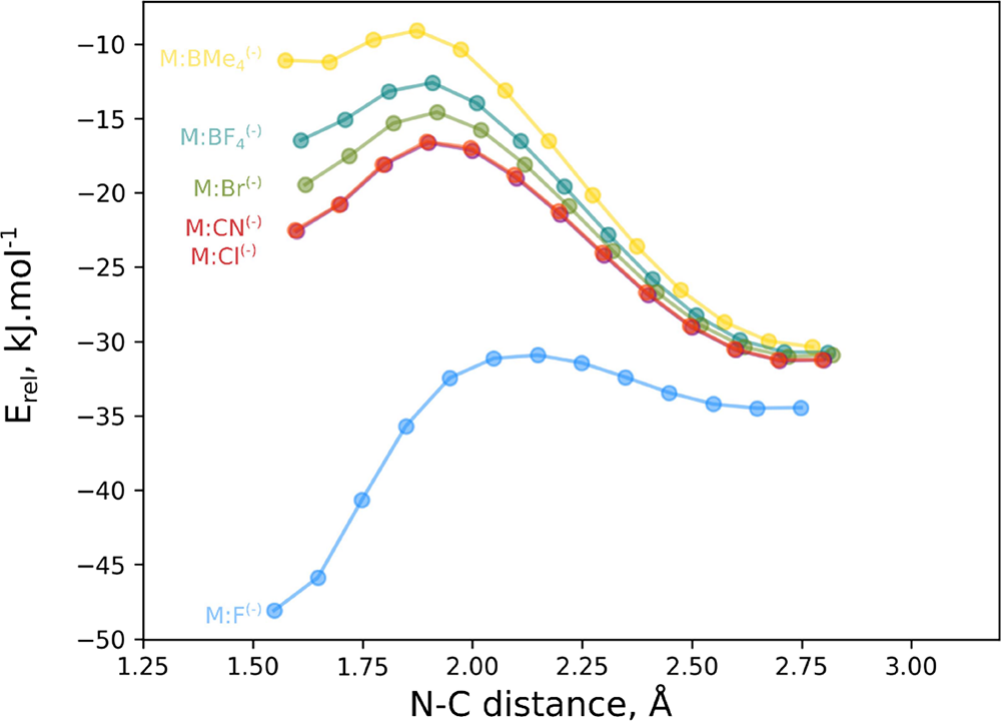
Relative energy vs N–C distance in the approach scan of the melamine:anion + CO_2_ systems.

**Figure 7:**
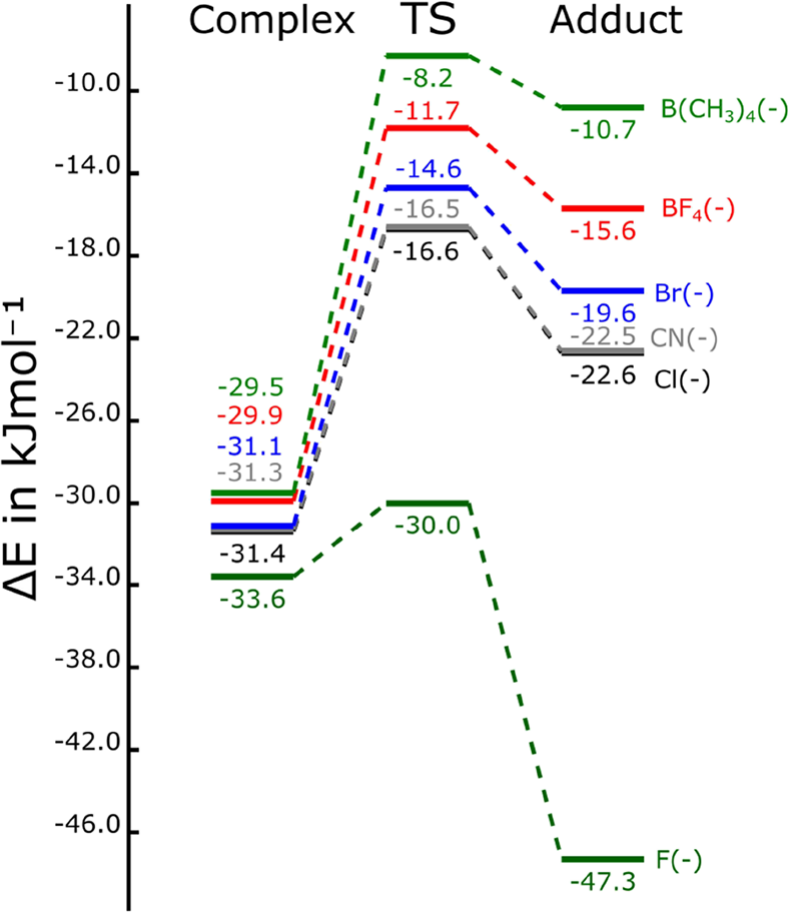
Energetic profile of the reaction between melamine:anion complexes and CO_2_.

The approaching scans of the melamine:anions and CO_2_ (Figure 6) show the presence of a second minima with short N–C distances corresponding to the adduct. The simplified energetic profile of the reaction of the melamine:anion complexes with CO_2_ including the adducts and the connecting TSs is shown in Figure 7.

It is interesting to notice that all the stationary points found are more stable than the entrance channel, sum of isolated melamine:anion, and CO_2_ energies. Only in one case (melamine:F^(−)^ complex), the resulting adduct is more stable than the noncovalent complex. In the rest of the cases, the noncovalent complexes are more stable than the adducts. The barrier of the transformation from the complex to the adducts decreases as the stability of the adduct increases, in agreement with Hammond's postulate. Thus, the barrier for the transformation between the noncovalent complex to the adduct in the most stable adduct melamine:F^(−)^–CO_2_ is only 3.6 kJ mol^–1^ while in the least stable one, melamine:B(CH_3_)_4_^(−)^–CO_2_, this barrier amounts to 21.2 kJ mol^–1^.

The intermolecular C–N distance in the TSs ranges from 1.86 to 2.13 Å, being larger as the barrier decreases. In fact, a linear relationship between the intermolecular C–N distance in the TS and the barrier is found with a negative slope (*R*^2^ = 0.97). In the adducts, the new N–C bond created ranges from 1.62 Å in the weakest adduct (melamine:B(CH_3_)_4_^(−)^–CO_2_) to 1.56 Å in the strongest one (melamine:F^(−)^–CO_2_). Also in this case, a linear correlation between these two parameters, N–C bond distance and the stability of the adduct, is found (*R*^2^ = 0.98).

**Figure 8:**
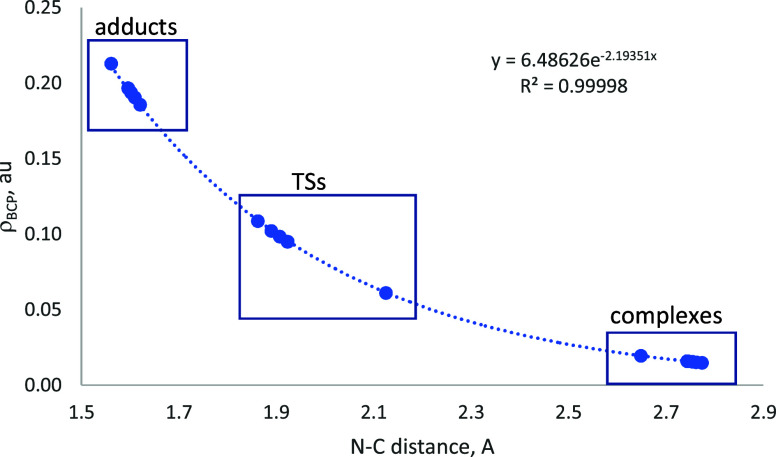
ρ_BCP_ vs. the interatomic N–C distance in the stationary points of the interaction of melamine with CO_2_. The exponential relationship found is shown.

The bond formation is also reflected in the electron density properties of the intermolecular N–C bonds. Thus, the representation of the ρ_BCP_ vs the interatomic N–C distances (Figure 8) clearly shows how this property increases from the noncovalent complexes to the TSs and finally to the adducts, following an exponential relationship in agreement with previous reports.^55−57^ In addition, the complexes present positive values of ∇^2^ρ_BCP_ and H_BCP_, in the TS, ∇^2^ρ_BCP_ remains positive and H_BCP_ becomes negative, and in the adducts, both properties are negative for all the systems studied here.

### Coupling the REG Method with an Energy Decomposition Scheme

**Figure 9:**
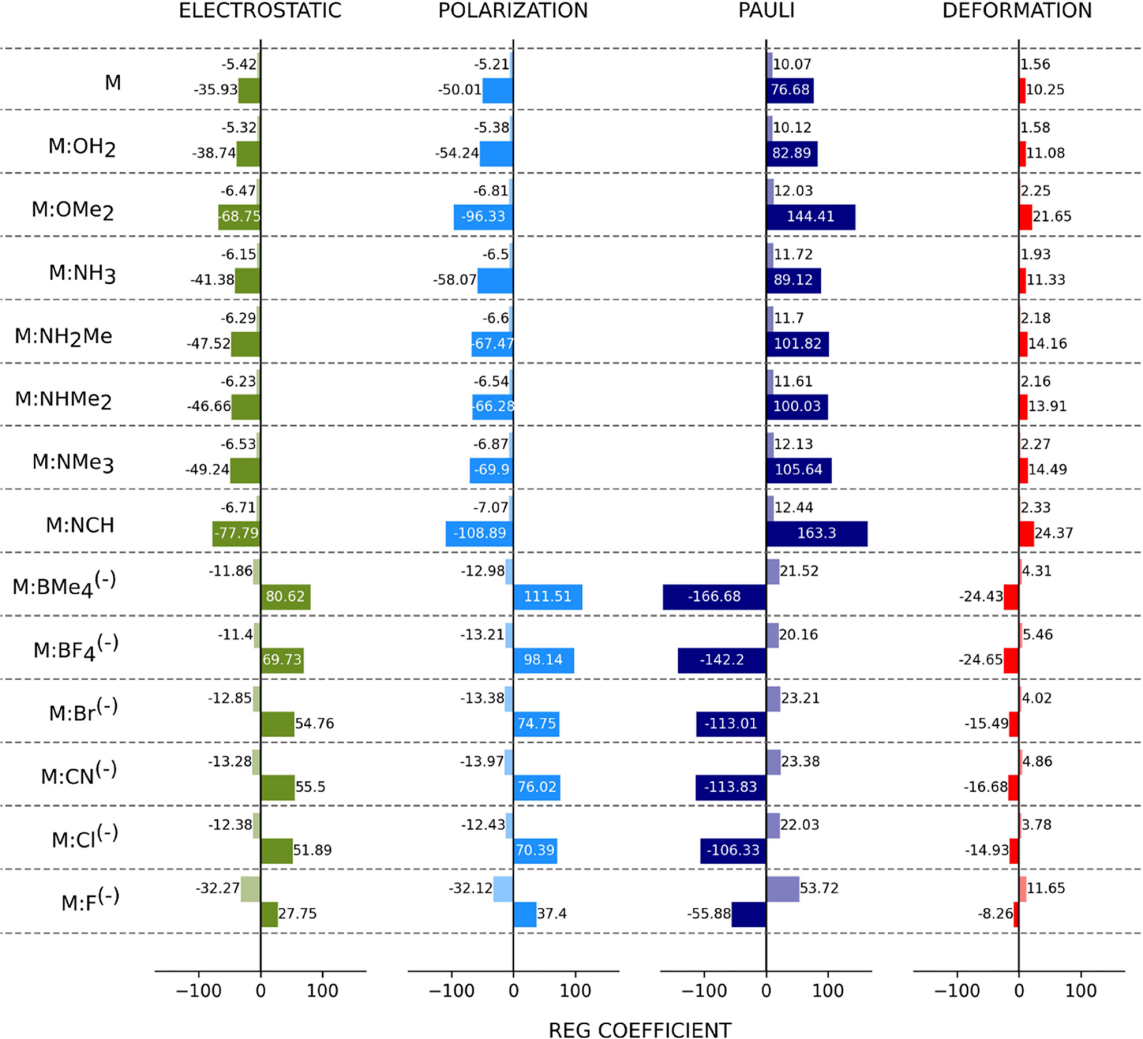
REG results for the different systems. The lighter bar (the first one to appear for each contribution) corresponds to barrier 1, and the second one, the darker, corresponds to barrier 2. A table with all of the values and the Pearson coefficients can be found in the SI.

In order to rationalize the importance of the different energetic terms in the CO_2_ capture by the melamine systems, the energy of the scan points was decomposed into deformation, electrostatic, Pauli repulsion, and polarization (eq 6), as described in the Computational methods section. Five functions are obtained: *E*_tot_(*d*_N–C_), *E*_def_(*d*_N–C_), *E*_elec_(*d*_N–C_), *E*_Pauli_(*d*_N–C_), and *E*_polar_(*d*_N–C_), with *d*_N–C_ being the coordinate that perturbs the system. Based on the evolution of the energy gradient along *d*_N–C_, the neutral systems present only one barrier. However, as already mentioned, a change in the energy evolution can be observed around 2.0 Å. For that reason, these scans were split into two parts. This splitting enables to improve the correlations. In the case of anionic systems, two barriers are observed: one from 2.75 Å to the TS, and one from the TS to the adduct. The REG results are listed in Figure 9.

**Figure 10:**
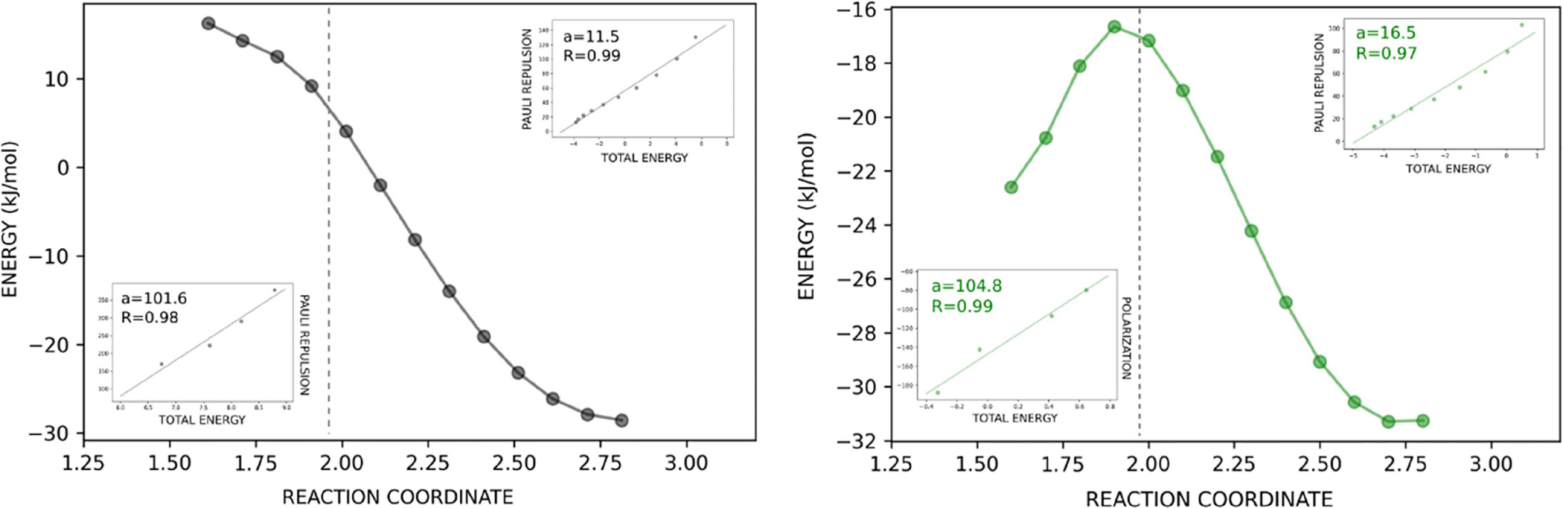
Illustration of the energy profile and REG results (insets) for the M:NH_3_–CO_2_ and M:Cl(−)–CO_2_ systems. The insets show the correlation of the Pauli repulsion (M:NH_3_–CO_2_) and the polarization and Pauli repulsion [M:Cl(−)–CO_2_]correlations with the energy along the reaction coordinate.

As it can be observed, in Figures 9 and 10, the energy contribution controlling the barriers in the neutral systems is the Pauli repulsion. This repulsion is directly related to the decrease in the distance between CO_2_ and the melamine system. It can be observed that the electrostatic and the polarization contributions have similar coefficients in Barrier 1, with the polarization being more important than the electrostatic term in Barrier 2 and its contribution against the barrier. In the case of the anionic systems, it can be seen that the reduction of the energy and thus the stabilization of the adduct (Barrier 2) are ruled by the polarization term, followed by the electrostatic energy. In this particular case, the Pauli repulsion term goes against the barrier. In the two barriers, the deformation energy has the same trend as the Pauli repulsion but to a lesser extent.

### Effect of an External Electric Field

The results obtained in the previous section indicate that polarization is the most important term in the formation of the adduct between melamine and CO_2_; here we explore the effect of an external electric field on the melamine:CO_2_ dimer formation. The electric field is applied in the three spatial directions and points toward positive and negative values of each axis with the system oriented, as indicated in Table 2. Adducts have been obtained only when the electric field is applied in the *Z* axes with positive values larger than 0.0027 au. Noncovalent complexes and adducts coexist with electric fields between 0.0027 and 0.0120 au. Stronger electric field yield only the adduct. As the electric field increased, the adduct became more stable following a linear correlation between these two properties (eq 7).
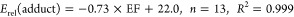
7

**Table 2:**
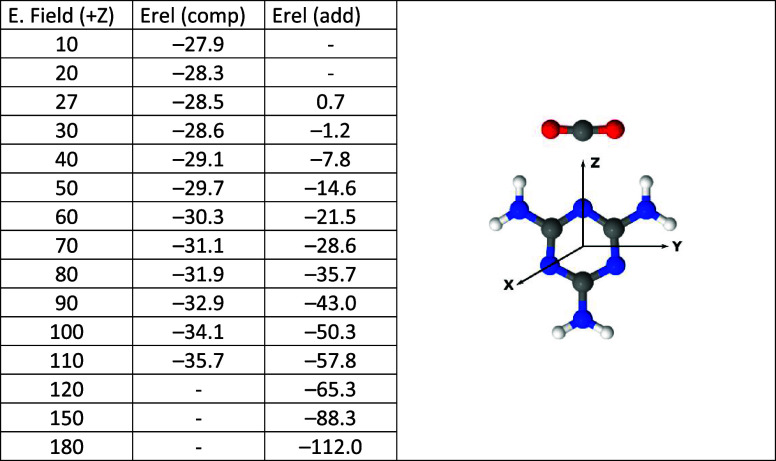
Electric Fields (10^4^ au) That Yield the Melamine-CO_2_ Adduct and Relative Energy of the Complexes and Adduct (kJ mol^–1^); the Orientation of the Melamine:CO_2_ System with Respect to the Cartesian Axes Is Shown

where *E*_rel_ is in kJ mol^–1^ and EF represented the electric field in au multiplied by 10^4^.

**Figure 11:**
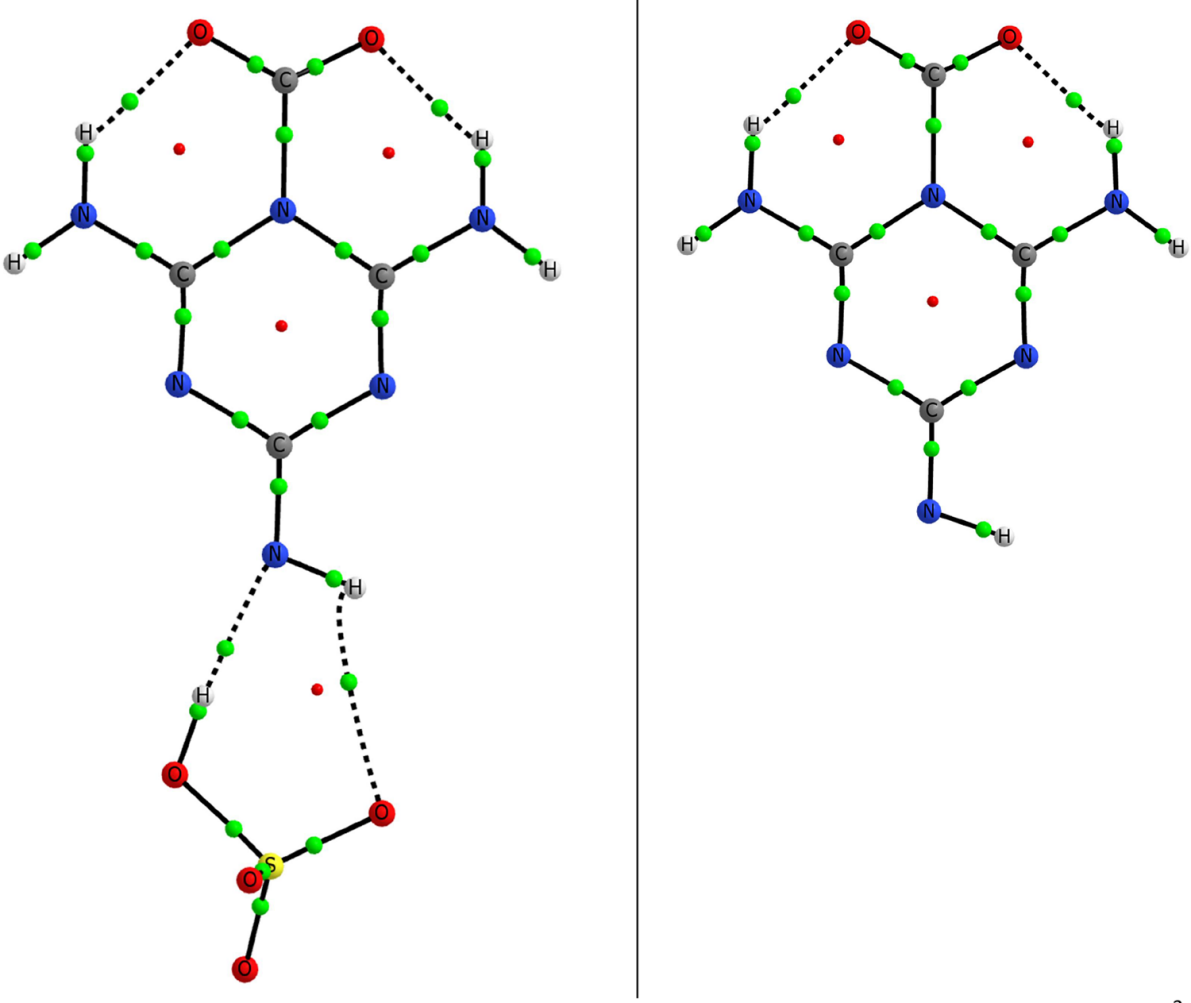
Molecular graph of the unique stationary points found for the melamine:SO_4_(^2–^) and melamine(^−^) adducts with CO_2_. The proton is transferred spontaneously on the melamine:SO_4_(^2–^) system upon the addition of the CO_2_.

These results are in good agreement with those of the energy partition discussed in the previous section. We recover here the electric field interpretation of FLP reactivity. A FLP system is able to activate a third molecule because it creates a particular electric field that polarizes the molecule.^58−61^ In fact, if the polarization of the studied systems is increased as in the case of the melamine:SO_4_(^2–^) and melamine(^−^) (Figure 11), the only stationary points obtained in the interaction with CO_2_ are the adducts with short C–N distances (1.53 and 1.54 Å, respectively).

## Conclusions

The capture of CO_2_ by melamine interacting with electron donors (neutral and anions) was studied by means of DFT computational methods. All the systems are able to form noncovalent complexes between melamine and CO_2_. Only the “melamine:anion” systems can form adducts with CO_2_ as well. The importance of the different energy terms, evaluated with the NCI-EDA method, has been analyzed along the reaction path with the REG method. This analysis indicates that polarization is the main energetic component in the formation of the melamine:CO_2_ adducts. These results are confirmed by calculating the effects of external electric fields on the melamine:CO_2_ complex.

## Supporting Information Available

Molecular graph, electronic energy, and Cartesian coordinates of the stationary points; relative energy vs N–C distance in the melamine + CO_2_ and melamine:anion + CO_2_ systems; properties of the ortho and para complexes; and REG results (PDF)
