# Supplementary material for: Capture of CO2 by Melamine Derivatives: A DFT Study Combining the Relative Energy Gradient Method with an Interaction Energy Partitioning Scheme
Source: J Phys Chem A. 2024 Feb 13;128(7):1288–96. doi: 10.1021/acs.jpca.3c08412 (PMC10895662; doi:10.1021/acs.jpca.3c08412)
Supplement: Supplementary file 1 — jp3c08412_si_001.pdf [file jp3c08412_si_001.pdf]

Capture of CO_2 by Melamine Derivatives: A DFT Study Combining the Relative Energy Gradient Method with an Interaction Energy Partitioning Scheme

Maxime Ferrer,[a,b] Ibon Alkorta,[*a] José Elguero,[a] Josep M. Oliva-Enrich[c]

[a] Instituto de Química Médica (CSIC), Juan de la Cierva, 3, E-28006 Madrid, Spain

[b] PhD Program in Theoretical Chemistry and Computational Modeling, Doctoral School, Universidad Autónoma de Madrid, 28049 Madrid, Spain

[c] Instituto de Química-Física Blas Cabrera (CSIC), Serrano, 119, E-28006 Madrid, Spain

*Author to whom correspondence should be addressed: e-mail: ibon@iqm.csic.es

Index

Pg. S2-S10	**Table S1:** Molecular graphs, energies (in Hartree) and Cartesian coordinates (in Å) of the monomers in vacuum. Level of theory: B3LYP-D3(BJ)/aug-cc-pvtz
Pg S11-S19	**Table S2:** Molecular graphs, energies (in Hartree) and Cartesian coordinates (in Å) of the complexes in vacuum. Level of theory: B3LYP-D3(BJ)/aug-cc-pvtz
Pg S20-S23	**Table S3:** Molecular graphs, energies (in Hartree) and Cartesian coordinates (in Å) of the transition states in vacuum. Level of theory: B3LYP-D3(BJ)/aug-cc-pvtz
Pg S24-S28	**Table S4:** Molecular graphs, energies (in Hartree) and Cartesian coordinates (in Å) of the adducts in vacuum. Level of theory: B3LYP-D3(BJ)/aug-cc-pvtz
Pg S29	**Figure S1:** Relative energy vs. N-C distance in the approach scan of the melamine:CO_2 complex.
Pg S30-S31	**Table S5:** Properties of the ortho and para complexes for the different system studied at B3LYP-D3(BJ)/aug-cc-pvtz.
Pg S32-S33	**Table S6:** REG results

<u>Table S1:</u> Molecular graphs, energies (in Hartree) and Cartesian coordinates (in Å) of the monomers in vacuum. Level of theory: B3LYP-D3(BJ)/aug-cc-pvtz

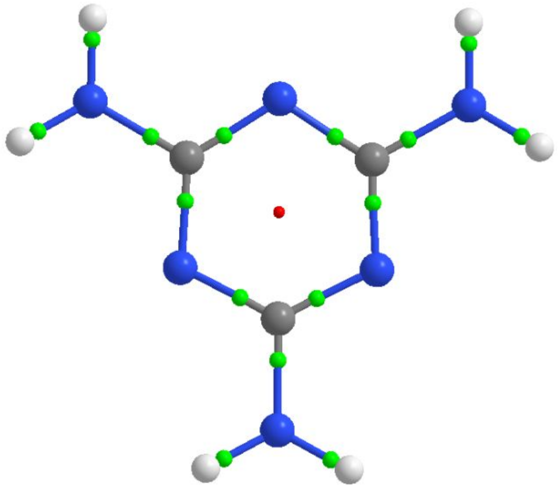	**M**<br>**E=-446.705321390H; FreqIm=0**<br>C,-0.1659371034,-1.2849096978,0.0276222703<br>C,-1.0298400843,0.7861379373,0.0272528593<br>C,1.1957685185,0.4987357856,0.0281009297<br>N,-2.1058555512,1.6075940814,0.0061505221<br>H,-3.0175166488,1.2097045352,0.1313745742<br>H,-1.9623436249,2.5918651243,0.1315851393<br>N,-0.3393066088,-2.6274866698,0.0061691857<br>H,0.4610759436,-3.2180810697,0.131496102<br>H,-1.2635084451,-2.9953956014,0.1311285028<br>N,2.4451422555,1.0199227094,0.0062197813<br>H,2.5563984471,2.0084161031,0.1311994937<br>H,3.2258998822,0.4035691478,0.1312908549<br>N,0.1762961396,1.3652624131,0.0304812671<br>N,1.0942480386,-0.8353900666,0.030667038<br>N,-1.2705771585,-0.5299867319,0.0302444795
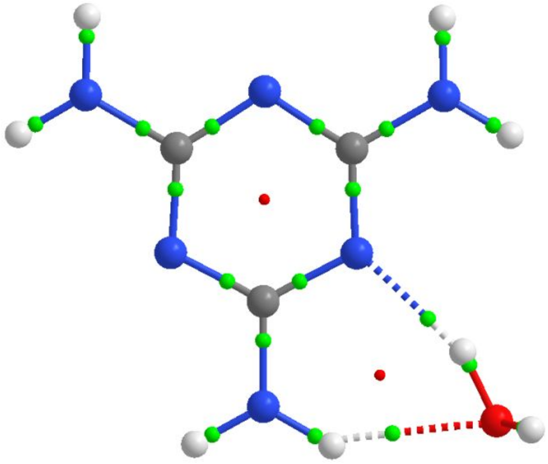	**M:OH$_2$**<br>**E=-523.188683347H; FreqIm=0**<br>C,-1.7946986775,-0.8514264191,0.0678128173<br>C,-0.2411436878,0.7820748263,-0.1077609899<br>C,0.3879072723,-1.3716772507,-0.0669607659<br>N,0.0724808982,2.097601552,-0.2046966528<br>H,-0.633617147,2.7759268409,0.0147850613<br>H,1.038643209,2.3533241251,-0.1140206321<br>N,-3.0827144737,-1.2222804056,0.1785583602<br>H,-3.2956650199,-2.1980649092,0.2606831409<br>H,-3.8048868488,-0.5181355711,0.2184880462<br>N,1.3658102634,-2.3022918708,-0.1180173392<br>H,2.3227695895,-2.0042088923,-0.1098673296<br>H,1.1291958596,-3.2656871361,0.0257841986<br>N,0.7693284621,-0.0885107994,-0.1386643749<br>N,-0.8684581032,-1.8155145509,0.0427098847<br>N,-1.5389651864,0.4691704767,-0.0101950511<br>O,-4.0942035049,1.5941499544,0.11269334<br>H,-4.3812398755,1.9667982925,-0.7251667241<br>H,-3.1305100292,1.4210927375,0.0165990105

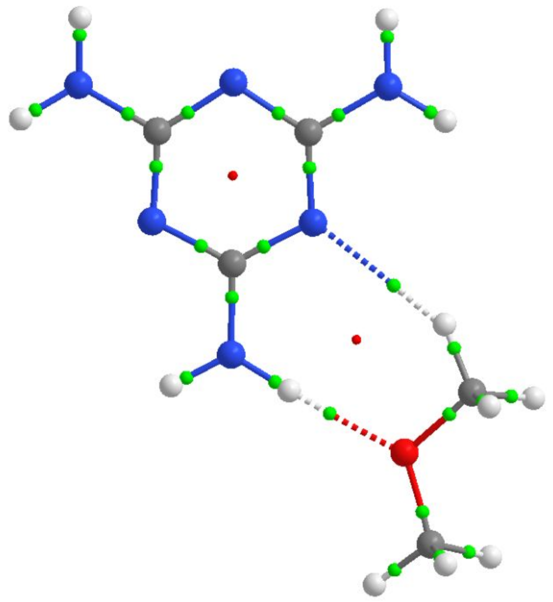	**M:O(CH_3)$_2$**<br>**E=-601.815245192H; FreqIm=0**<br>C,-0.7007142063,-0.8662897722,-0.029202102<br>C,-1.6117558609,1.1499773754,-0.4357359021<br>C,-2.9164742942,-0.5567815284,0.2049690972<br>N,-1.4704083312,2.4609396073,-0.7705358484<br>H,-0.605093968,2.7423598089,-1.1932940839<br>H,-2.3052097447,2.972029169,-0.9927166603<br>N,0.3730333022,-1.6736782287,0.0295443316<br>H,0.2309929823,-2.6245207459,0.3129195747<br>H,1.299457987,-1.3278334581,-0.1810209578<br>N,-4.1493847142,-1.0517798999,0.4807302114<br>H,-4.9052660404,-0.4017233723,0.5910542939<br>H,-4.2097330013,-1.9593250907,0.9036604189<br>N,-2.8492886245,0.7332841147,-0.1489494769<br>N,-1.8887727548,-1.4037091345,0.289576365<br>N,-0.5008797933,0.4095483583,-0.4008831026<br>O,3.0421443833,-0.5280893682,-0.5211044708<br>C,4.3661268041,-1.0197641984,-0.5259653677<br>H,4.3131887632,-2.104962953,-0.5834240978<br>H,4.9235537976,-0.6417490017,-1.3901988803<br>H,4.8983303514,-0.7328345657,0.3880029488<br>C,2.9708165421,0.887626489,-0.4505271782<br>H,3.4609340124,1.342907654,-1.3181986366<br>H,1.9149520025,1.1469011656,-0.4411112152<br>H,3.4531038159,1.2561090055,0.4615967397
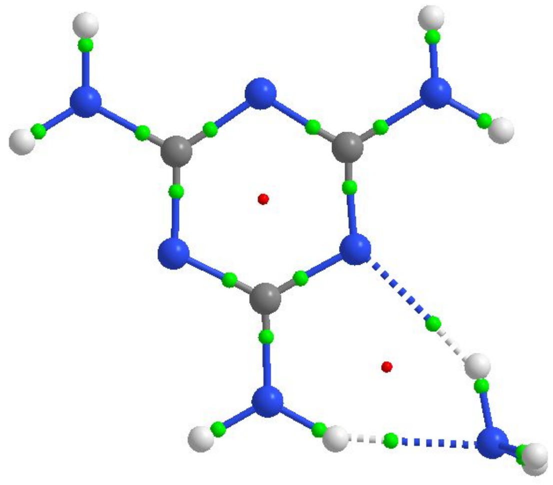	**M:NH_3**<br>**E=-503.308201473H; FreqIm=0**<br>C,1.7534406971,-0.8397743721,-0.020000649<br>C,0.1961407988,0.7907176508,0.0049786983<br>C,-0.4355638874,-1.3605518157,-0.021257558<br>N,-0.1273441998,2.1094037599,0.0568693581<br>H,0.593920106,2.7816201185,-0.1272766642<br>H,-1.0836221361,2.3605124611,-0.1147263372<br>N,3.0421121837,-1.221647073,-0.0314031892<br>H,3.2433787423,-2.2035350024,-0.0225413744<br>H,3.7794433432,-0.5252671893,0.0038147034<br>N,-1.4193004746,-2.2919630001,-0.059884859<br>H,-2.3637313729,-1.9936902147,0.0978129675<br>H,-1.1692576001,-3.2535874677,0.0765913935<br>N,-0.8197466585,-0.0770889507,-0.004265559<br>N,0.8228571326,-1.8054878641,-0.0233890908<br>N,1.4965349479,0.4827715492,-0.0085210841<br>N,4.45715485,1.4122859259,0.0882842342<br>H,4.8162491704,1.8485723347,0.928465927<br>H,3.4450928912,1.5330738135,0.0709983341<br>H,4.8512214661,1.9024983362,-0.7051762493

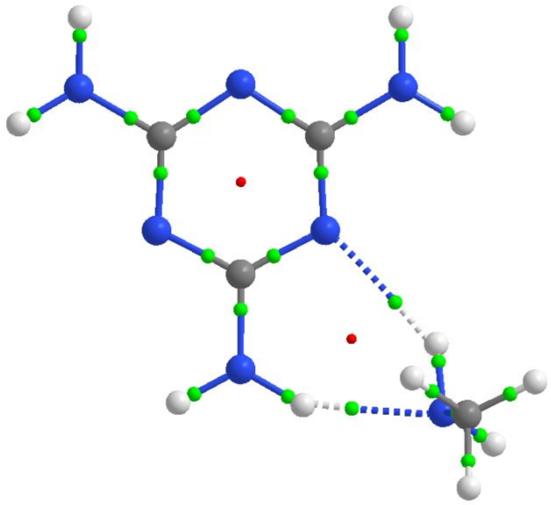	**M:NH_2CH_3**<br>**E=-542.627131148H; FreqIm=0**<br>C,-1.2715109298,1.1219253299,-0.1956939365<br>C,0.1042743051,-0.6644387548,-0.1961794868<br>C,0.9495711281,1.3992194589,0.0367754087<br>N,0.2868259611,-2.010519344,-0.2420997902<br>H,-0.4777930498,-2.5805080481,-0.552748602<br>H,1.2245960592,-2.3479238021,-0.3594213355<br>N,-2.5091025833,1.6395533824,-0.273050838<br>H,-2.6089511603,2.6344869061,-0.2031200339<br>H,-3.3114462565,1.0231670812,-0.3818523155<br>N,2.0219152281,2.2178683278,0.168707575<br>H,2.9117254507,1.8056350354,0.3786567706<br>H,1.8581035332,3.1851662407,0.3769662423<br>N,1.1989446672,0.0848128786,-0.035602344<br>N,-0.2516938699,1.9771017547,-0.0282569941<br>N,-1.1510232321,-0.2168251658,-0.291550886<br>N,-4.1910343698,-0.7814913482,-0.378957323<br>C,-4.5651896742,-1.1934038803,0.975606362<br>H,-3.8814389232,-0.727540123,1.6847487122<br>H,-5.5709599431,-0.8401302489,1.2036409891<br>H,-4.5379602759,-2.2757808468,1.1446885065<br>H,-4.7869141132,-1.21286728,-1.0732210999<br>H,-3.2273149515,-1.0421545537,-0.571875580
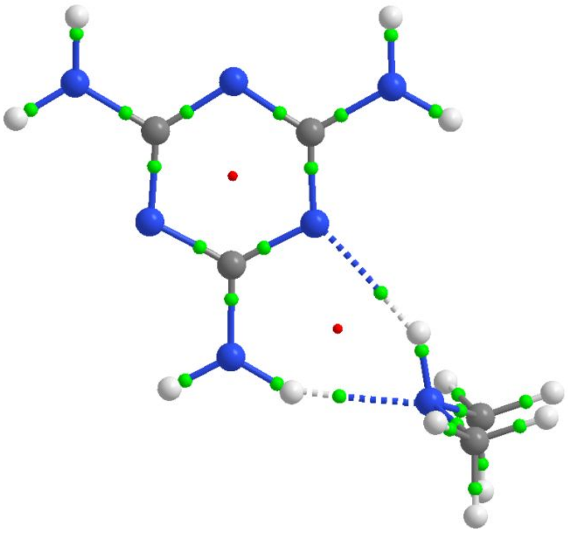	**M:$NH(CH_3)_2$**<br>**E=-581.951590177H; FreqIm=0**<br>C,-0.8133277075,1.2883912382,-0.0367757342<br>C,0.4279840885,-0.594939616,-0.0164953539<br>C,1.4336997828,1.4089613655,-0.0429041118<br>N,0.5102833059,-1.9503464872,0.0339331235<br>H,-0.3198530353,-2.4821032547,-0.150819417<br>H,1.4056559963,-2.3681978903,-0.1412229611<br>N,-2.014281663,1.8909905208,-0.0435406184<br>H,-2.0389194821,2.8931110736,-0.0393276459<br>H,-2.8627583893,1.3274808329,-0.0128181495<br>N,2.5680095016,2.1493896767,-0.0834262802<br>H,3.4442481546,1.6868250344,0.0713841295<br>H,2.4944164758,3.1401400936,0.0537469548<br>N,1.5824965558,0.0773680859,-0.0274582714<br>N,0.2751223047,2.0718618502,-0.0419722036<br>N,-0.7962179316,-0.0593709218,-0.026849333<br>N,-3.8575047076,-0.4092361059,0.0594128007<br>C,-4.5016976645,-0.8202348673,1.2972521793<br>H,-3.903872486,-0.4903663958,2.1463077672<br>H,-5.4859396368,-0.354102305,1.3750893774<br>H,-4.6422292928,-1.9085728949,1.375155591<br>C,-4.5385608843,-0.8869249974,-1.133799112<br>H,-5.5252894615,-0.4253631411,-1.207110501<br>H,-3.9674174462,-0.6037879214,-2.017485988<br>H,-4.6805326086,-1.9779007323,-1.147837498<br>H,-2.8851537689,-0.7075582405,0.0522652597

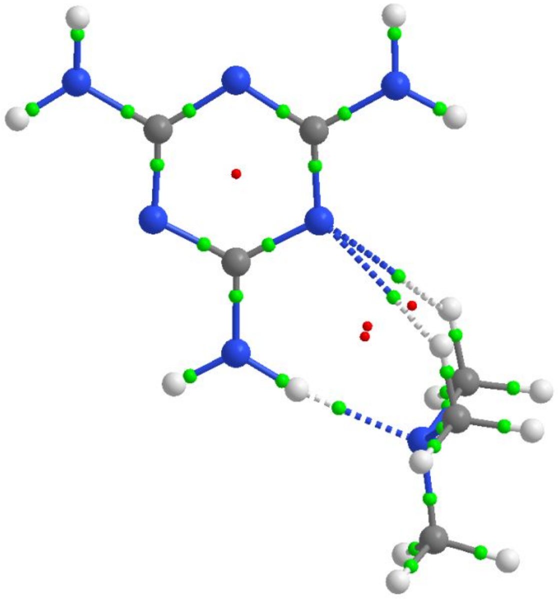	**M:N(CH$_3$)$_3$**<br>**E=-621.278267480H; FreqIm=0**<br>C,0.5289956865,1.1628927465,0.0332289489<br>C,-0.8234332618,-0.6374536126,0.0183925171<br>C,-1.7068631257,1.4225325174,0.032706017<br>N,-0.9870556975,-1.9868789368,-0.028149863<br>H,-0.1888857307,-2.5627602592,0.1656452636<br>H,-1.9047221535,-2.3495240818,0.1546719127<br>N,1.7627007744,1.6946793446,0.0393082721<br>H,1.8378171698,2.6945572566,0.032793841<br>H,2.5923952544,1.0977051934,0.0167196431<br>N,-2.7956827932,2.2317707785,0.0701930821<br>H,-3.6943437666,1.8213595644,-0.103409984<br>H,-2.6595039205,3.212576695,-0.0900730626<br>N,-1.9367220805,0.1027163412,0.0224930229<br>N,-0.5106555529,2.0132317742,0.0310455182<br>N,0.4306841913,-0.1791969028,0.0310279059<br>N,3.9799573186,-0.2676901419,-0.0341153599<br>C,3.6133546041,-1.0023702796,-1.23923043<br>H,2.5479693472,-1.223194675,-1.2107397413<br>H,3.8170741333,-0.3903307638,-2.1177083565<br>H,4.1756049228,-1.9444231324,-1.3366619351<br>C,5.3737331657,0.1473531386,-0.0588547179<br>H,5.5602497996,0.7625710233,-0.9390100709<br>H,5.5991389629,0.7380905302,0.828962133<br>H,6.0660890969,-0.7092105474,-0.0860397914<br>C,3.6663066056,-1.0356806023,1.1647277215<br>H,3.9101915151,-0.4491286965,2.050353056<br>H,2.6000225355,-1.2538343897,1.1770512447<br>H,4.2297399994,-1.9810218821,1.2103982131
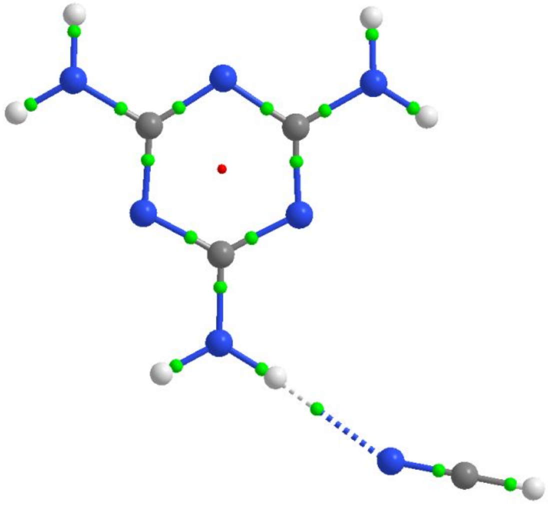	**M:NCH**<br>**E=-540.174985449H; FreqIm=0**<br>C,-2.2004566555,-0.5863523263,0.147255945<br>C,0.044227386,-0.5390552275,0.032380982<br>C,-1.1264135137,1.3780942553,0.0289172906<br>N,1.221518638,-1.1902903572,-0.0203184262<br>H,2.0897957283,-0.68115939,-0.0120526246<br>N,-3.3575789203,-1.287490301,0.2584977093<br>H,-4.2212164372,-0.7976828735,0.116132391<br>H,-3.3221040032,-2.280413644,0.120675878<br>N,-1.1592751442,2.7370078618,0.0172737074<br>H,-2.0450816036,3.1806957389,-0.1414585487<br>N,0.076973516,0.8015080942,-0.0114756034<br>N,-2.3044793579,0.7476470972,0.1008187982<br>N,-1.0660396951,-1.2888735308,0.1136643326<br>H,-0.3165598344,3.2208383018,-0.2321403465<br>N,4.0316348724,0.267854286,0.1057342551<br>C,4.7468267234,1.1512504243,0.2394269576<br>H,5.4113984949,1.9752166802,0.3644522614<br>H,1.2175029961,-2.1885317293,0.0694709809

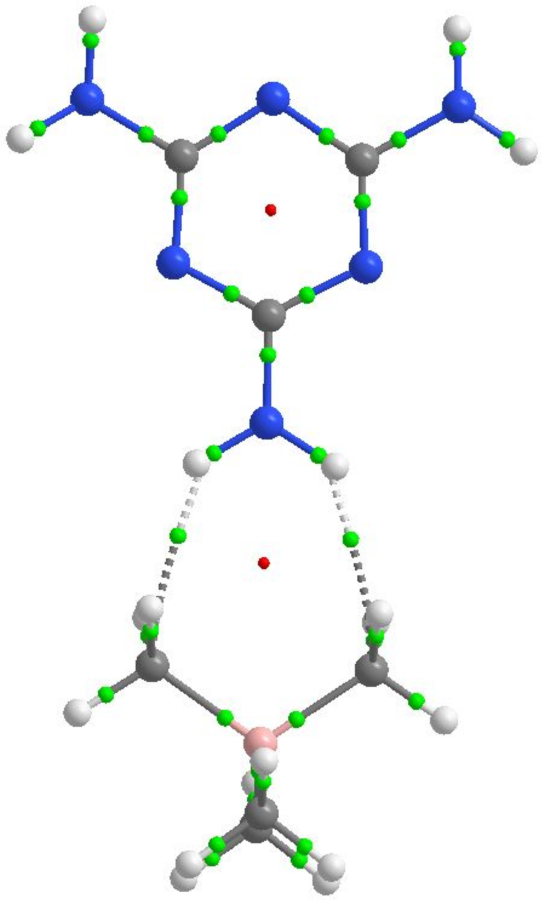	**M:B(CH$_3$)$_4$$^{(-)}$**<br>**E=-631.375992629H; FreqIm=0**<br>C,0.,0.,-0.2070290563<br>C,-0.0520293156,1.1176724603,-2.162080453<br>C,0.0520293156,-1.1176724603,-2.162080453<br>N,-0.147868224,2.2980429872,-2.8486142842<br>H,0.0578210724,3.1296483369,-2.3237010751<br>H,0.1198388964,2.273157103,-3.8161015834<br>N,0.,0.,1.1245620166<br>H,0.0386285327,-0.8588082534,1.648043395<br>H,-0.0386285327,0.8588082534,1.648043395<br>N,0.147868224,-2.2980429872,-2.8486142842<br>H,-0.1198388964,-2.273157103,-3.8161015834<br>H,-0.0578210724,-3.1296483369,-2.323701075<br>N,0.,0.,-2.9005944635<br>N,0.0471375187,-1.1928344128,-0.836989852<br>N,-0.0471375187,1.1928344128,-0.836989852<br>B,0.,0.,5.1062129195<br>C,0.0524764072,-1.3584947276,4.1637307932<br>H,0.9438479978,-1.3877963,3.5193038959<br>H,0.0874827498,-2.2724018224,4.7711625274<br>H,-0.8335997493,-1.4560283182,3.518741217<br>C,-0.0524764072,1.3584947276,4.1637307932<br>H,0.8335997493,1.4560283182,3.5187412178<br>H,-0.9438479978,1.3877963,3.5193038959<br>H,-0.0874827498,2.2724018224,4.7711625274<br>C,-1.3484347371,-0.0521254631,6.0534530233<br>H,-1.4279329119,0.8269353129,6.7083238253<br>H,-2.2737136766,-0.0879400473,5.460887851<br>H,-1.3597393737,-0.9346591194,6.708389461<br>C,1.3484347371,0.0521254631,6.0534530233<br>H,1.3597393737,0.9346591194,6.708389461<br>H,1.4279329119,-0.8269353129,6.7083238253<br>H,2.2737136766,0.0879400473,5.4608878513

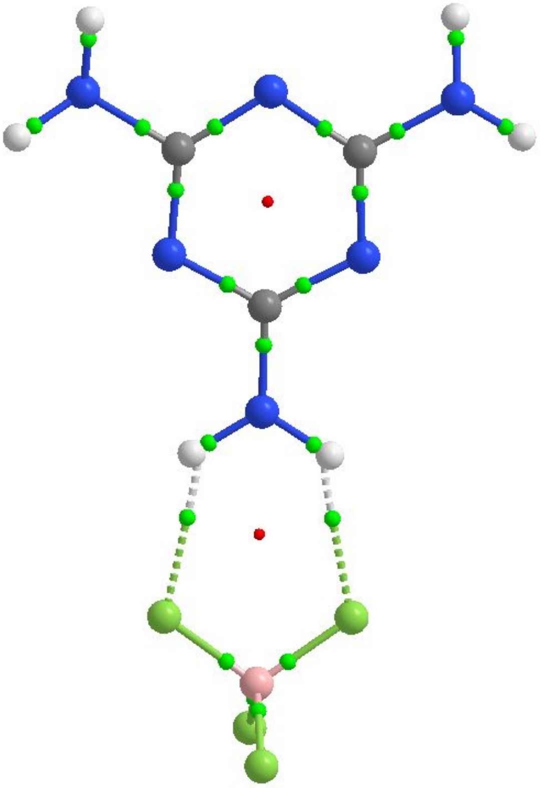	**$BF_4^{(-)}$**<br>**E=-871.449552083H; FreqIm=0**<br>C,0.999973,-0.005523,0.00018<br>C,2.953846,1.120977,-0.009841<br>C,2.963377,-1.115473,0.009787<br>N,3.638153,2.308695,-0.062375<br>H,3.108251,3.125066,0.18812<br>H,4.601046,2.273458,0.2214<br>N,-0.32648,-0.011309,0.000157<br>H,-0.862115,-0.864978,0.007681<br>H,-0.871228,0.836964,-0.004856<br>N,3.6579,-2.297364,0.062243<br>H,4.620271,-2.253654,-0.222197<br>H,3.135033,-3.118078,-0.188881<br>N,3.699046,0.005887,-0.000139<br>N,1.640801,-1.197328,0.001914<br>N,1.630604,1.191561,-0.001656<br>B,-3.743882,-0.000137,-0.000204<br>F,-2.906239,1.15048,0.003929<br>F,-4.529178,-0.000129,-1.15825<br>F,-2.914884,-1.156503,0.0102<br>F,-4.549164,0.006265,1.143898
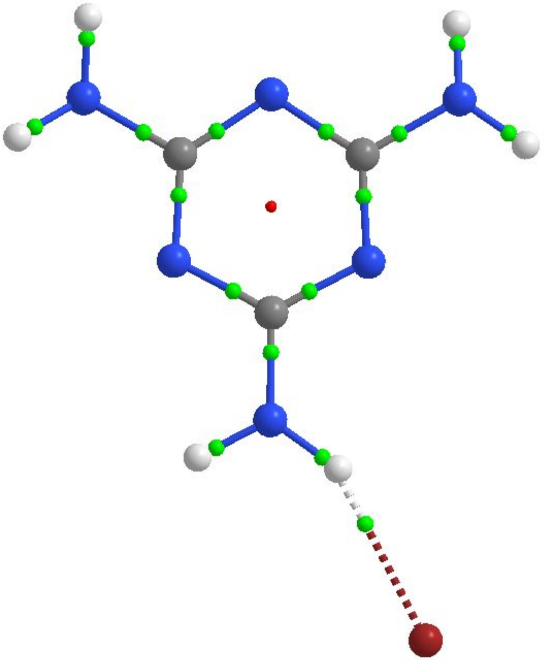	**M:$Br^{(-)}$**<br>**E=-3021.04387543H ; FreqIm=0**<br>C,-2.1011219982,-0.8191273523,0.051429719<br>C,0.1231192284,-0.452821604,-0.0926524311<br>C,-1.3191352098,1.2752612921,0.0038924455<br>N,1.3612728146,-0.927138576,-0.1667676702<br>H,1.4870326724,-1.921974197,-0.1340763083<br>H,2.2131282308,-0.3535834248,-0.1413556481<br>N,-3.165179166,-1.6892643703,0.0808707463<br>H,-4.025721775,-1.307989354,0.4326459914<br>H,-2.940539233,-2.6338626874,0.3405547494<br>N,-1.5489087768,2.6256815531,-0.0150289768<br>H,-0.7497093177,3.2044937909,0.1762093564<br>H,-2.4436003786,2.9296428967,0.3245020883<br>N,-0.056799352,0.8839755035,-0.0823091409<br>N,-2.4027909719,0.4860502735,0.0853019056<br>N,-0.8915500527,-1.3530649919,-0.030258804<br>Br,4.5579412857,-0.0725827521,0.0124619779

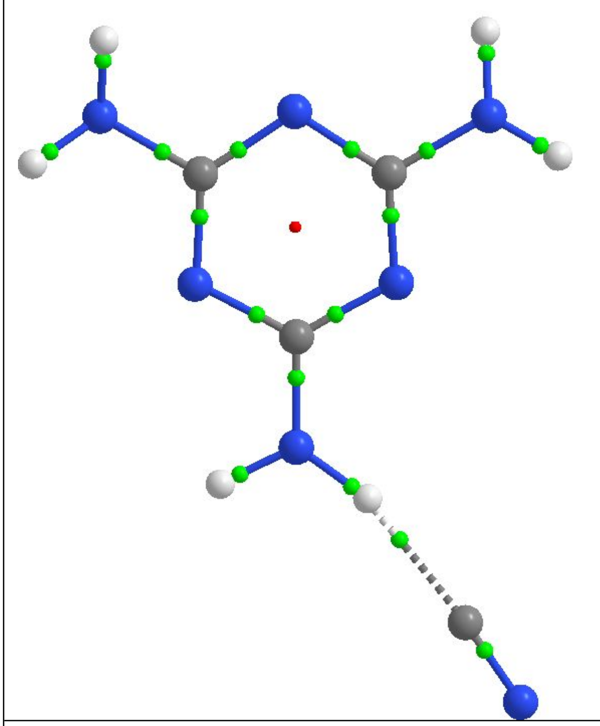	**M:CN$^{(-)}$**<br>**E=-539.6266266H; FreqIm=0**<br>C,-0.2561025727,-0.5368237563,-0.024683595<br>C,1.0576848496,1.2959990671,-0.003952496<br>C,1.9975007658,-0.7319820044,0.0140018867<br>N,1.1846128196,2.6612051274,-0.0457688482<br>H,0.3532546544,3.1763700099,0.187108454<br>H,2.0658395983,3.0316196793,0.2622483739<br>N,-1.4551202649,-1.1028432103,-0.042535050<br>H,-1.4747724987,-2.1070268187,-0.030717405<br>H,-2.3794135367,-0.6023330166,-0.028982934<br>N,3.1253645568,-1.5196567533,0.0773189717<br>H,3.9764365086,-1.0703795626,-0.2127724215<br>H,2.9932909117,-2.4731081065,-0.21279988<br>N,2.2020606821,0.5923192064,0.0189033332<br>N,0.8307750194,-1.3562007413,-0.0163329086<br>N,-0.1735175542,0.8114906365,-0.0177562582<br>C,-4.2334824268,-0.191722149,0.0140869869<br>N,-5.3855585122,-0.0093106077,0.0470867912
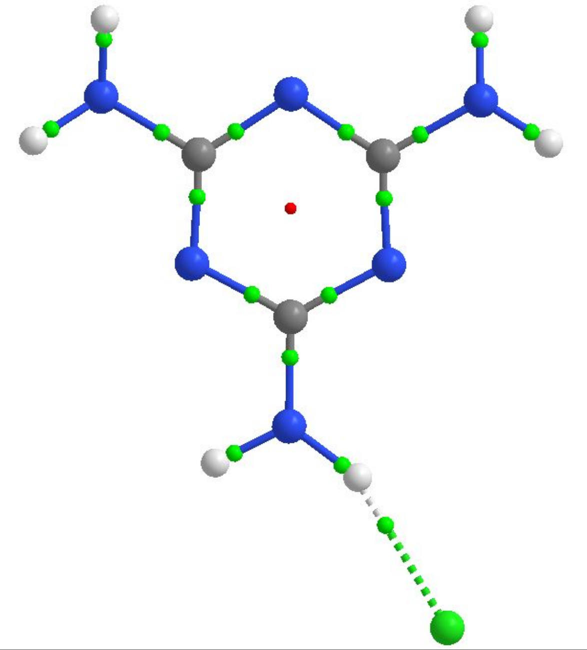	**M:Cl$^{(-)}$**<br>**E=-907.039618926H; FreqIm=0**<br>C,-2.0896648305,-0.8189109324,0.0481876705<br>C,0.1390694321,-0.4616489035,-0.0860009839<br>C,-1.2993793662,1.2716674476,0.0033315872<br>N,1.3731793402,-0.9415749375,-0.1522664762<br>H,1.4806266167,-1.9390642172,-0.1273044283<br>H,2.2440431138,-0.3796769895,-0.1309511587<br>N,-3.1591061417,-1.6854508969,0.0728651009<br>H,-4.0156587601,-1.2989834597,0.4292901224<br>H,-2.9371229232,-2.6283473347,0.3415944287<br>N,-1.5248308165,2.6243210835,-0.0181901683<br>H,-0.7233289236,3.1972522303,0.1818727841<br>H,-2.417423187,2.9293268009,0.3263611196<br>N,-0.0389552746,0.8768121594,-0.0768090015<br>N,-2.3875777006,0.4874176779,0.080619177<br>N,-0.8830681135,-1.357567759,-0.0280704279<br>Cl,4.3366355345,-0.0018759694,-0.0191093458

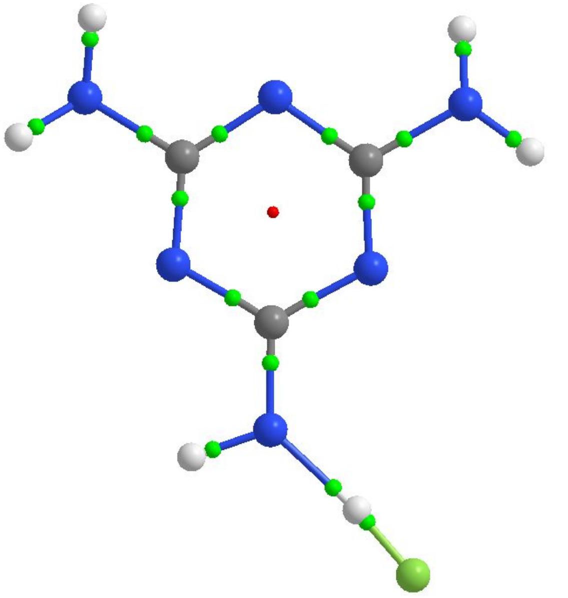	**M:$F_4^{(-)}$**<br>**E=-546.655212086H; FreqIm=0**<br>C,-1.3207899876,0.6108544497,-0.0114919874<br>C,0.3843266928,-0.8967198163,0.0715699178<br>C,0.8662581703,1.2759996934,-0.0861588944<br>N,0.8188140503,-2.2081491369,0.2053026308<br>H,0.1181639755,-2.8863474538,-0.0452673561<br>H,1.7399667015,-2.3739129728,-0.1641802789<br>N,-2.6007536176,0.881417216,-0.0193615792<br>H,-2.7361892941,1.8823877157,-0.0863802014<br>H,-3.7629858526,0.0421720126,0.0273044603<br>N,1.8143556518,2.2856095463,-0.2148204988<br>H,2.7170160689,2.0379647973,0.1558891266<br>H,1.4699877681,3.1905925396,0.0609957152<br>N,1.3603631416,0.0286799534,-0.0085302717<br>N,-0.3964533448,1.6388178803,-0.0813015471<br>N,-0.9133921243,-0.6987275102,0.0655800936<br>F,-4.672622346,-0.4705021385,0.0519384254
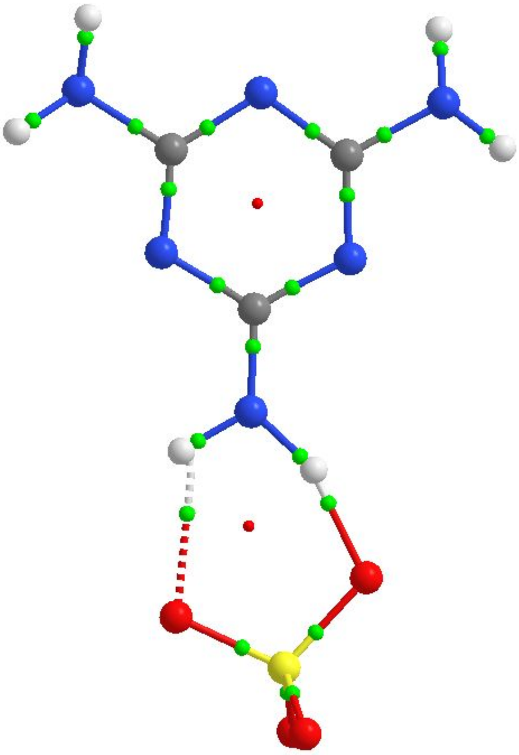	**M:$SO_4^{(2-)}$**<br>**E=-1145.96017397H; FreqIm=0**<br>C,-1.133915,-0.120331,0.008377<br>C,-3.213396,-1.050586,-0.019265<br>C,-3.006705,1.169094,0.016078<br>N,-4.028767,-2.179951,-0.082513<br>H,-3.558725,-3.01174,0.237362<br>H,-4.942708,-2.029528,0.312775<br>N,0.159861,-0.240577,0.011752<br>H,0.947788,0.513011,0.018093<br>H,0.609059,-1.151018,-0.00204<br>N,-3.599817,2.427419,0.07385<br>H,-4.520066,2.454526,-0.333509<br>H,-2.976318,3.160945,-0.223485<br>N,-3.86256,0.128165,-0.007439<br>N,-1.696562,1.133093,0.015956<br>N,-1.918276,-1.255066,-0.002709<br>S,3.421676,-0.010424,-0.002395<br>O,2.698031,-1.341398,-0.009513<br>O,4.245945,0.143124,-1.242102<br>O,2.346132,1.097627,0.016483<br>O,4.265028,0.119388,1.227094

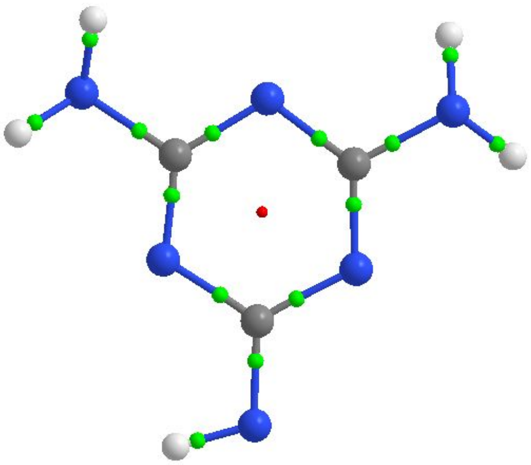	**M(-)**<br>**E=-446.116961879H; FreqIm=0**<br>C,1.1845348647,-0.5919417741,-0.0047352754<br>C,-0.4958335421,0.965560329,0.0960242303<br>C,-1.0391520216,-1.1883285033,-0.0953247181<br>N,-0.9049380424,2.2939766301,0.2481630275<br>H,-0.1863018195,2.946428392,-0.0222491504<br>H,-1.8097711483,2.4732584294,-0.1569212187<br>N,2.4509897197,-0.8798900702,-0.0094206816<br>H,2.5189103812,-1.8924034359,-0.0893481815<br>N,-2.0256191429,-2.1695440435,-0.243045713<br>H,-2.907502211,-1.8965369765,0.1609038616<br>H,-1.7021443578,-3.0814133233,0.0381857759<br>N,-1.5035100501,0.0722818685,0.0005491931<br>N,0.2059994619,-1.5895955686,-0.091502238<br>N,0.791148344,0.7383939013,0.0886000871

Table S2: Molecular graphs, energies (in Hartree) and Cartesian coordinates (in Å) of the complexes in vacuum. Level of theory: B3LYP-D3(BJ)/aug-cc-pvtz

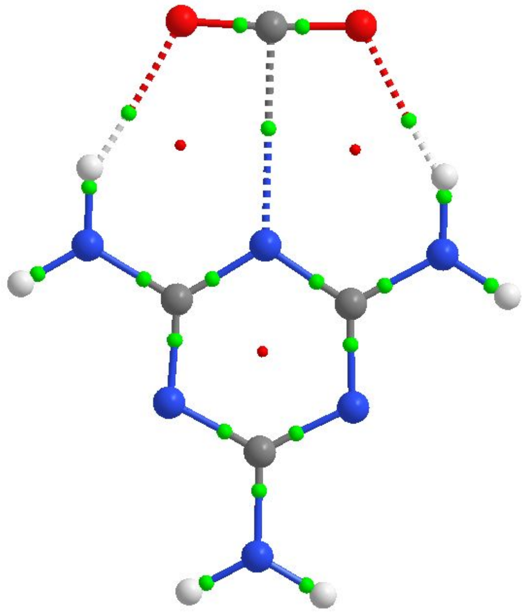	**Complex M**<br>**E=-635.380924691H; FreqIm=0**<br>C,2.3489464409,-0.0013418355,0.0115235814<br>C,0.4061277429,-1.1261418938,0.0589980493<br>C,0.4068715741,1.1256312113,0.0305310609<br>N,-0.2653885012,-2.2967736674,0.1008605499<br>H,0.2535129101,-3.1515387871,0.0349649634<br>H,-1.2668007209,-2.2969565904,0.0453248985<br>N,3.7020963916,-0.0024555276,-0.0392286663<br>H,4.1859568328,0.8676532342,0.0807821717<br>H,4.1853398875,-0.8695969088,0.10273947<br>N,-0.263861099,2.2973759102,0.0425662653<br>H,-1.2653769313,2.2968683761,-0.0111743793<br>H,0.2555475439,3.1500105324,-0.043784273<br>N,-0.3225945838,0.0000414063,0.0498809813<br>N,1.7429529293,1.1910852315,0.0102651851<br>N,1.7421519458,-1.193020455,0.0403733029<br>C,-3.1532261648,-0.0002944724,-0.065019661<br>O,-3.196801941,1.1594330942,-0.0818142739<br>O,-3.1976052569,-1.1600408582,-0.052852225
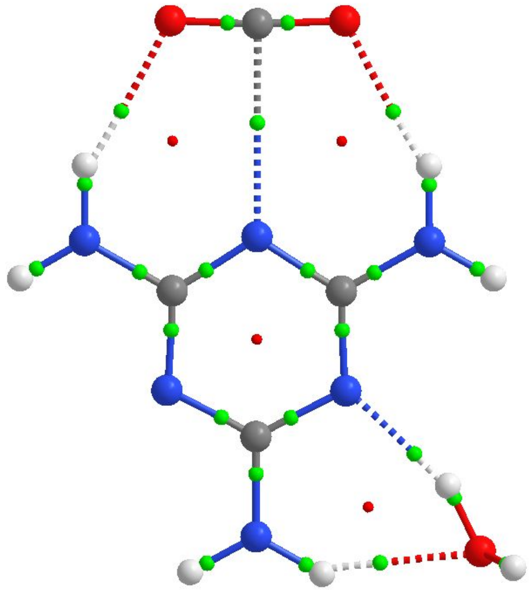	**Complex M:OH_2**<br>**E=-711.864153984H; FreqIm=0**<br>C,-1.822316283,-0.8134219319,0.0506079098<br>C,-0.2400474664,0.8031342151,0.0031637342<br>C,0.3554750439,-1.3680445324,-0.0450047998<br>N,0.0932981388,2.1132285204,-0.0150337981<br>H,-0.6191393602,2.793620079,0.1724055963<br>H,1.0602970494,2.3656016667,0.0800423234<br>N,-3.1182088865,-1.167600666,0.1043805962<br>H,-3.347917458,-2.142768744,0.1296315307<br>H,-3.8310617887,-0.4547392334,0.1577330914<br>N,1.315420655,-2.3125976621,-0.1034718952<br>H,2.2815249802,-2.0431393249,-0.1063041015<br>H,1.0477260946,-3.2777679558,-0.0716180476<br>N,0.7582018577,-0.0860450635,-0.0418496231<br>N,-0.9115870198,-1.7910805495,0.0034449675<br>N,-1.5440653827,0.5047355221,0.0468401922<br>C,3.4957430741,0.6683530714,0.0322800079<br>O,3.8455416616,-0.4354627176,-0.0452174173<br>O,3.2258605581,1.7942460108,0.1131101997<br>O,-4.0896514907,1.6587967691,0.1504495743<br>H,-4.3456546949,2.0793923012,-0.6747028719<br>H,-3.1252612823,1.4804862253,0.0762388307

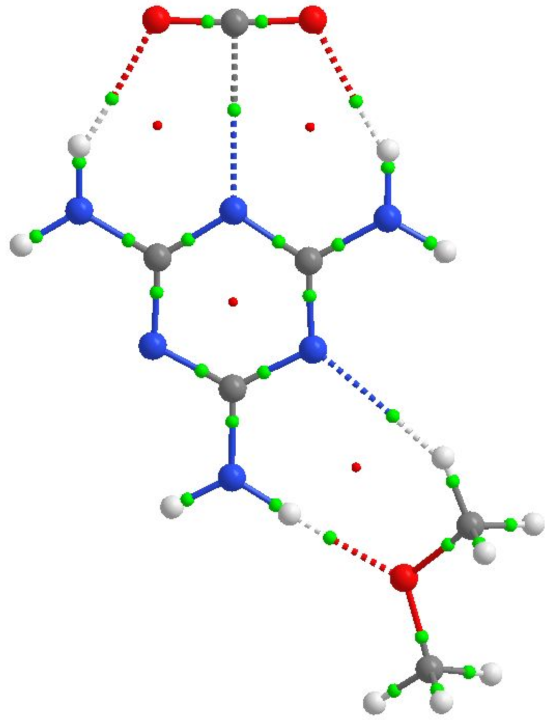	**Complex M:$O(CH_3)_2$**<br>**E=-790.490836280H; FreqIm=0**<br>C,-0.7004349719,-0.8666021034,-0.047195719<br>C,-1.6051342707,1.1634427568,-0.4046526687<br>C,-2.9200015627,-0.5666873,0.1779279184<br>N,-1.4575752385,2.477716617,-0.7042157337<br>H,-0.5726008679,2.7793337563,-1.0662307356<br>H,-2.279863001,3.0237362475,-0.8865604444<br>N,0.3724235524,-1.6753437876,-0.0019871296<br>H,0.2273740066,-2.6345849176,0.2499512366<br>H,1.3001808062,-1.3253806468,-0.2000883244<br>N,-4.1484744824,-1.0697699385,0.4369020798<br>H,-4.9302907834,-0.4454623729,0.5088271429<br>H,-4.2150403193,-2.010268537,0.777119453<br>N,-2.8478802522,0.7361117509,-0.1400264143<br>N,-1.8906711013,-1.4130144997,0.245467727<br>N,-0.4968542985,0.4190238063,-0.3799882953<br>O,3.0438144674,-0.5254114813,-0.5209104134<br>C,4.3655491986,-1.0233914243,-0.5282731754<br>H,4.3077038374,-2.1069107667,-0.6081423161<br>H,4.9296457572,-0.6306105104,-1.381476197<br>H,4.8937773385,-0.7577409794,0.394364139<br>C,2.9788366666,0.8889754243,-0.4231453152<br>H,3.4742520726,1.3587285788,-1.2799621095<br>H,1.9241166777,1.1527749784,-0.4124544521<br>H,3.459227818,1.2374326372,0.4977940792<br>C,-5.1206674511,2.4122648143,-0.2849177913<br>O,-4.5034603865,3.287156492,-0.7332687238<br>O,-5.8114337917,1.5916507658,0.1584509235
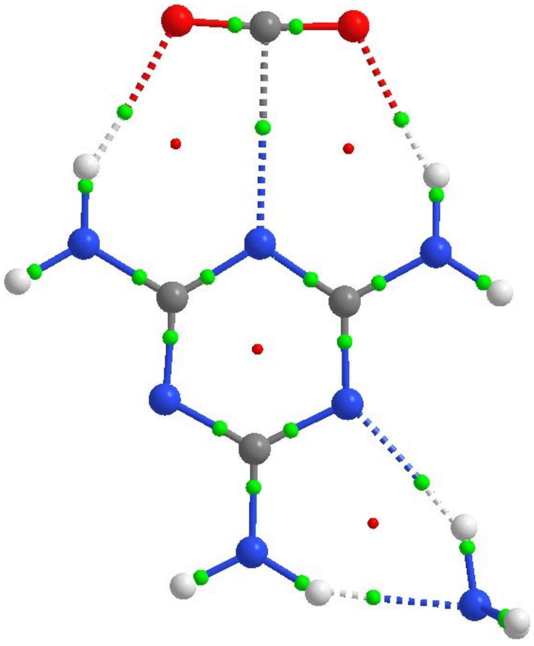	**Complex M:NH_3**<br>**E=-691.983778584H; FreqIm=0**<br>C,1.7295632302,-1.0156925569,-0.0497406199<br>C,0.3264054323,0.7495258081,0.027626722<br>C,-0.4971781887,-1.3444506138,0.005743212<br>N,0.1273670807,2.0877263759,0.0896615475<br>H,0.9119023903,2.6944641096,-0.0546650848<br>H,-0.8059824613,2.4437547467,-0.0063176628<br>N,2.9780759474,-1.5100757214,-0.097312104<br>H,3.0913145608,-2.5059452618,-0.108748406<br>H,3.7750467282,-0.881751864,-0.0906239176<br>N,-1.5543205379,-2.1859536704,-0.002546891<br>H,-2.4792487472,-1.8189204577,0.1233378902<br>H,-1.3817014309,-3.1712266501,0.0606363156<br>N,-0.7642143525,-0.0284460771,0.0405673237<br>N,0.7177960469,-1.8953098567,-0.0343937869<br>N,1.5921423532,0.3240094304,-0.0225901907<br>C,-3.3981500768,1.0090358807,0.090572823<br>O,-3.8611699298,-0.0488455924,0.2062654131<br>O,-3.0183005694,2.0999345808,-0.0238002453<br>N,4.6268324221,0.9846219267,-0.0363204751<br>H,5.0553199078,1.3837162049,0.7897152351<br>H,3.6303742342,1.1981616914,-0.0161680317

	H,5.0348029602,1.4391695673,-0.8438690656
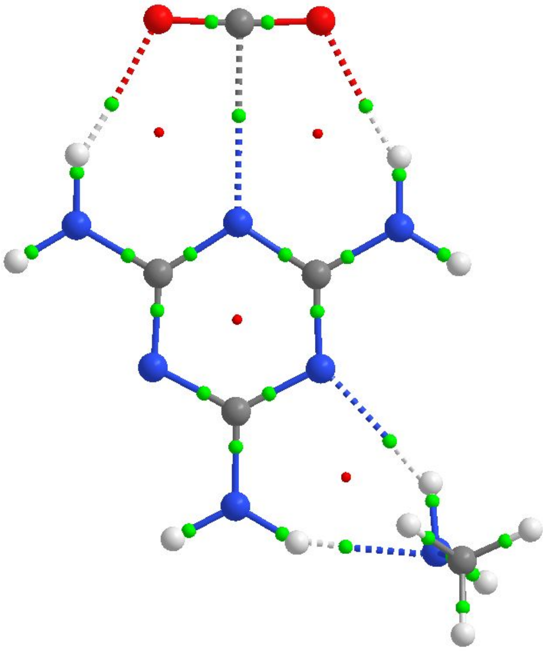	**Complex M:NH_2CH_3**<br>**E=-731.302716102H; FreqIm=0**<br>C,1.7806423404,-0.9997328741,-0.07499659<br>C,0.4099015259,0.7924223397,-0.0782086746<br>C,-0.4514294872,-1.2843315302,0.0127196506<br>N,0.2354191277,2.1356932815,-0.0818814465<br>H,1.0296993294,2.7191342815,-0.2639764083<br>H,-0.6918994242,2.5034221745,-0.1912619084<br>N,3.0201756825,-1.5167979645,-0.1024272588<br>H,3.1156985133,-2.5141158394,-0.0696820626<br>H,3.8277428376,-0.8982825314,-0.1371612964<br>N,-1.5236739595,-2.105653956,0.054221827<br>H,-2.4409932971,-1.7158934059,0.1664901979<br>H,-1.3683950684,-3.0896028661,0.1648585146<br>N,-0.694601279,0.0364290811,-0.0170530129<br>N,0.753056914,-1.8587319848,-0.0073666311<br>N,1.6671778963,0.342114601,-0.1171321912<br>C,-3.3083052029,1.1226084484,-0.0044414028<br>O,-3.7895487913,0.0806627914,0.1677456879<br>O,-2.9097784616,2.1991715208,-0.1765200344<br>N,4.7082942163,0.8983388862,-0.0108095484<br>H,5.3484046336,1.3539864442,-0.6478165067<br>H,3.7596165224,1.1729962675,-0.2523906937<br>C,4.9981250871,1.251085283,1.3806511724<br>H,4.2682426312,0.7618409163,2.0251592841<br>H,5.9854906716,0.8806178528,1.6567335946<br>H,4.9661730618,2.3257878326,1.5925365877
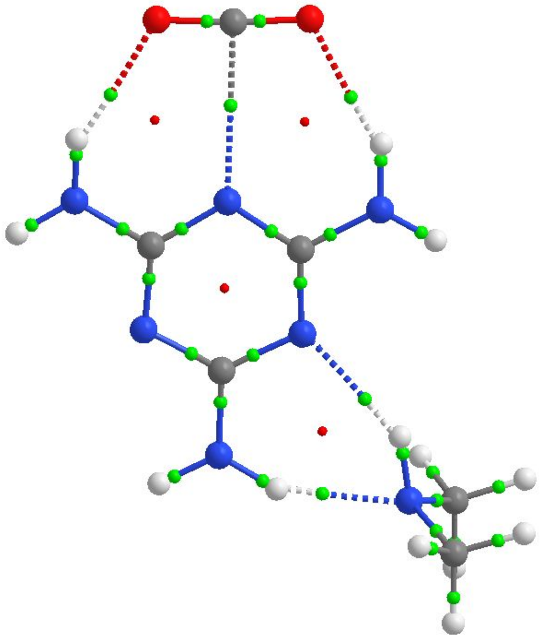	**Complex M:$NH(CH_3)_2$**<br>**E=-770.627164057H; FreqIm=0**<br>C,1.5430527514,-1.3019234008,0.2487833173<br>C,0.4804075396,0.6711494359,-0.0211387533<br>C,-0.6955228756,-1.2471845449,0.0116112583<br>N,0.5185809262,2.0226937092,-0.09883143<br>H,1.4111534344,2.4714560264,-0.178696683<br>H,-0.3159154558,2.5171711035,-0.356255147<br>N,2.6802137633,-1.9959710503,0.420860142<br>H,2.6154932784,-2.9924408315,0.5092994064<br>H,3.5686666238,-1.5014820291,0.4912010271<br>N,-1.8767273359,-1.8967931817,-0.077464492<br>H,-2.7291728089,-1.3701698346,-0.122100570<br>H,-1.8928944019,-2.8856714062,0.084748903<br>N,-0.7246231843,0.0911643571,-0.1007837976<br>N,0.3961637923,-1.9939012305,0.1911426044<br>N,1.6450255052,0.0379236663,0.1459392988<br>C,-3.113277132,1.5509402856,-0.5212348546<br>O,-3.7694637359,0.6025743556,-0.3911250126<br>O,-2.5322946155,2.5455400883,-0.6649526266<br>N,4.7051530745,0.1439318595,0.513304454<br>H,3.7698586911,0.520780679,0.380797955<br>C,5.2664174821,0.6176853686,1.7690175853

	H,4.563823117,0.4233018465,2.5787782413<br>H,6.1912660876,0.0801169101,1.9874180108<br>H,5.4999931272,1.6927072006,1.7641102865<br>C,5.5363074497,0.4443079418,-0.6421657949<br>H,6.4780727634,-0.1037588013,-0.5735294781<br>H,5.0261707096,0.1257975349,-1.5507666892<br>H,5.7800899589,1.512851302,-0.7381060201
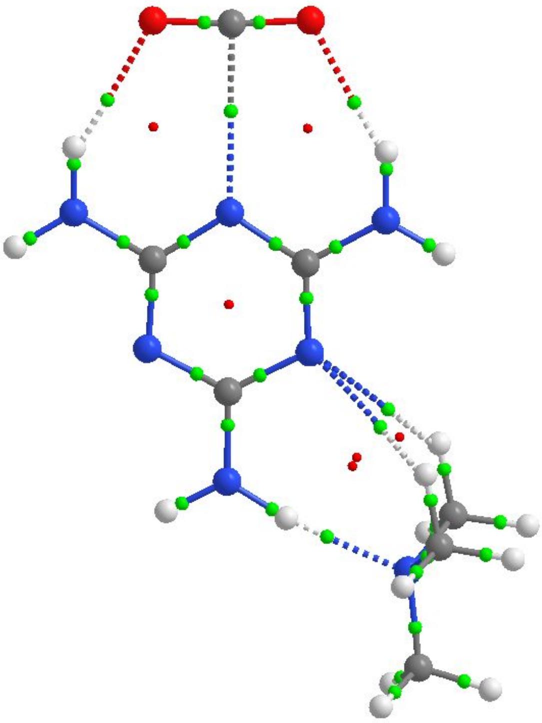	**Complex M:N(CH_3)$_3$**<br>**E=-809.953881811H; FreqIm=0**<br>C,0.4440084492,-1.1639073393,-0.0444403957<br>C,-0.795934293,0.7166338763,0.0148449926<br>C,-1.8046137597,-1.2935688201,-0.030426624<br>N,-0.8736358086,2.0684201002,0.0818203631<br>H,-0.033301563,2.5972083242,-0.0560402105<br>H,-1.7671107572,2.5082828664,-0.0421169492<br>N,1.6433293563,-1.7678169869,-0.0702254998<br>H,1.6585887561,-2.7703505722,-0.085465622<br>H,2.5075437463,-1.2215769526,-0.0486886252<br>N,-2.9341758813,-2.0373731563,-0.066759766<br>H,-3.821010182,-1.5890900921,0.0707874568<br>H,-2.8494546376,-3.0324457438,0.0200434926<br>N,-1.9536670298,0.0408786073,0.0057195147<br>N,-0.643962093,-1.9508709445,-0.049863171<br>N,0.4266259692,0.181046468,-0.0160769747<br>C,-4.4802005618,1.3095766348,0.0329192071<br>O,-5.0378829561,0.2995778143,0.1593214625<br>O,-4.0045057544,2.3609567172,-0.0927999961<br>N,3.97372809,0.0560617678,0.0083283325<br>C,3.6661695525,0.7875855881,1.2317861288<br>H,2.6158035297,1.0726329374,1.2209863033<br>H,3.8426232861,0.1472126106,2.095827256<br>H,4.2852958221,1.6920060402,1.3399105573<br>C,5.3397085838,-0.4437110339,0.0082185484<br>H,5.4976557823,-1.0869063778,0.8738413892<br>H,5.5188901013,-1.0289574101,-0.8936384435<br>H,6.0834978593,0.3682051502,0.0438657967<br>C,3.6947303314,0.8658642404,-1.1715629192<br>H,3.893914889,0.283911121,-2.0712637357<br>H,2.6434869298,1.1479622247,-1.1676157816<br>H,4.3135992428,1.7763333404,-1.2048450866

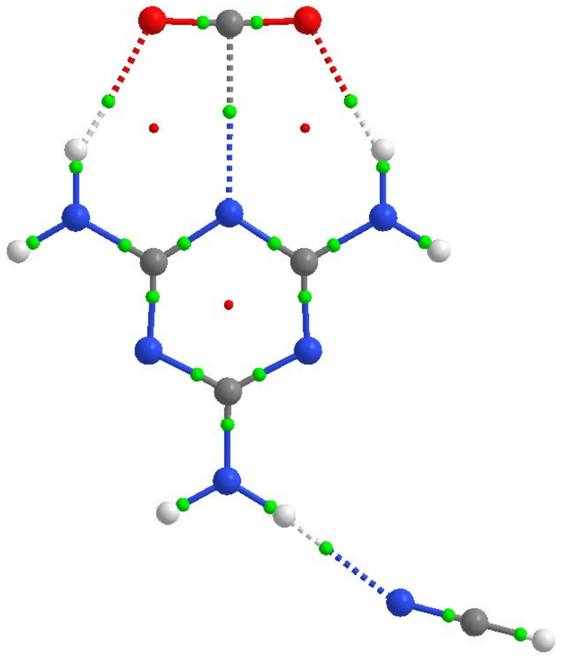

Complex M:NCH
E=-728.850635210H; FreqIm=0
C,2.33519128,0.0226414865,-0.0366528145
C,0.4021445266,-1.1190796563,0.1080474298
C,0.3789383735,1.1288607423,0.0110679292
N,-0.2573916669,-2.2957493082,0.2143675085
H,0.267439074,-3.1445341583,0.1208293249
H,-1.2583159071,-2.3061654323,0.1511301762
N,3.6786910155,0.0292607228,-0.1134365866
H,4.1890967216,0.8966047609,-0.1326750462
H,4.1640312439,-0.8460804768,-0.062193131
N,-0.305994266,2.2974608235,0.0201036109
H,-1.3052649562,2.2777050828,-0.0670107607
H,0.2009153622,3.1406382076,-0.1721257928
N,-0.3408508074,-0.001028795,0.0873508027
N,1.7097797972,1.2089166646,-0.0564604689
N,1.7342309544,-1.1743469212,0.0469669619
N,5.4635327561,2.6465687405,-0.1192216932
C,5.7363995329,3.756208506,-0.0563680898
H,5.9890038069,4.7901619004,0.0023519697
C,-3.1634781881,-0.0362564577,-0.061616383
O,-3.2212168816,1.1210499034,-0.1305352556
O,-3.1995925589,-1.1947149187,0.0002431758

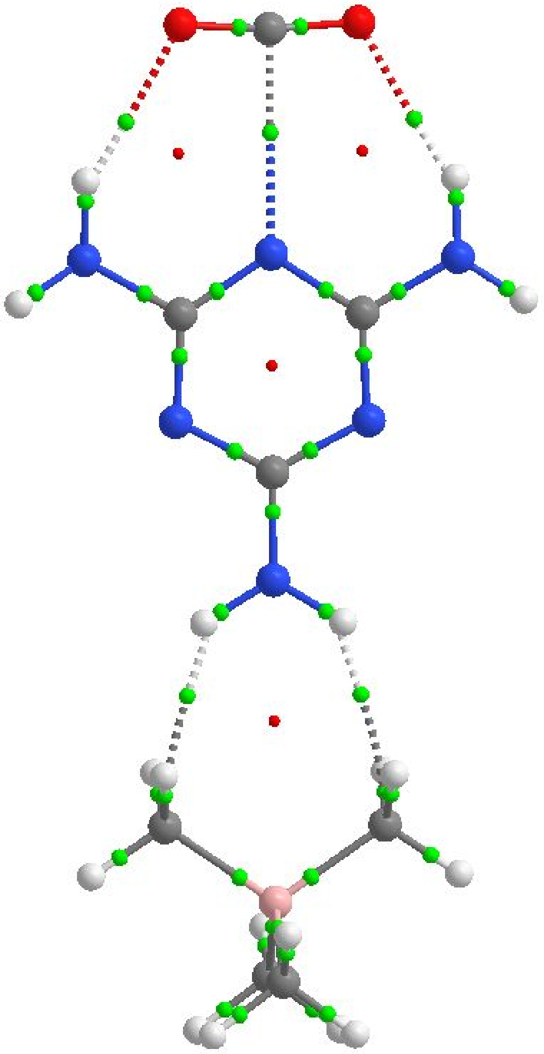

Complex M:$B(CH_3)_4^{(-)}$
E=-820.052255034H; FreqIm=0

C,0.,0.,0.0609775969
C,-0.0477988941,1.1223106456,-1.8936112388
C,0.0477988941,-1.1223106456,-1.8936112388
N,-0.139433085,2.3025962616,-2.5725941638
H,0.0345529749,3.1333450564,-2.0361189625
H,0.1052754424,2.2989112434,-3.5461694968
N,0.,0.,1.391729064
H,0.0342243431,-0.8589876903,1.9155947031
H,-0.0342243431,0.8589876903,1.9155947031
N,0.139433085,-2.3025962616,-2.5725941638
H,-0.1052754424,-2.2989112434,-3.546169496
H,-0.0345529749,-3.1333450564,-2.036118962
N,0.,0.,-2.6327470622
N,0.0415161265,-1.1927647967,-0.5688568444
N,-0.0415161265,1.1927647967,-0.5688568444
C,0.,0.,-5.4067219308
O,-0.2622201177,-1.1296488107,-5.469160513
O,0.2622201177,1.1296488107,-5.4691605132
B,0.,0.,5.3635766093
C,0.0459788917,-1.3590911648,4.4214758151
H,0.9374423464,-1.393302144,3.7774102935
H,0.0765685737,-2.2727308377,5.0294452163
H,-0.8407131248,-1.453029899,3.7768022863
C,-0.0459788917,1.3590911648,4.4214758151
H,0.8407131248,1.453029899,3.7768022863
H,-0.9374423464,1.393302144,3.7774102935
H,-0.0765685737,2.2727308377,5.0294452163
C,-1.348739365,-0.0456553784,6.310487139
H,-1.4241976725,0.8337412976,6.9653359173
H,-2.2740829303,-0.0770331283,5.7177680095
H,-1.3644688547,-0.9280859607,6.9654196007
C,1.348739365,0.0456553784,6.310487139
H,1.3644688547,0.9280859607,6.9654196007
H,1.4241976725,-0.8337412976,6.9653359173
H,2.2740829303,0.0770331283,5.7177680095

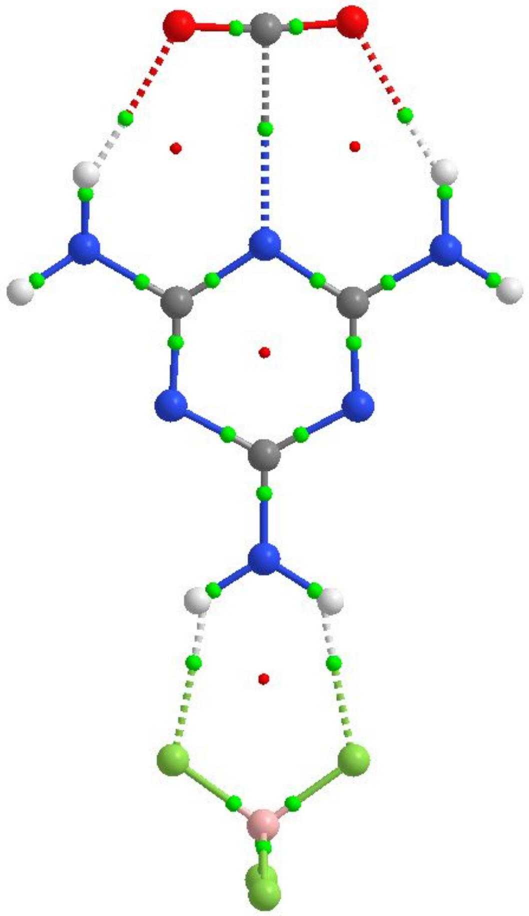	**Complex M:$BF_4^{(-)}$**<br>**E=-1060.12598542H; FreqIm=0**<br>C,0.1122124729,0.0001035763,-0.0001933532<br>C,-1.8457560734,1.1218039837,0.0488173021<br>C,-1.8456775526,-1.1217444886,-0.048969374<br>N,-2.52795124,2.3035388767,0.1437656149<br>H,-1.9897854637,3.1301579395,-0.0461024785<br>H,-3.4953169132,2.2956507717,-0.125440869<br>N,1.4377480687,0.0001548929,-0.0002782192<br>H,1.9784952002,-0.8500515663,-0.0347305505<br>H,1.9784164605,0.850403253,0.0342912648<br>N,-2.5277877686,-2.3035336159,-0.143890699<br>H,-3.4951209426,-2.2957483415,0.125441671<br>H,-1.989526833,-3.1301080235,0.045905032<br>N,-2.5867783804,0.0000013573,-0.0000409702<br>N,-0.5233148757,-1.1933649512,-0.042434757<br>N,-0.5233999726,1.1935257457,0.0421244259<br>C,-5.3485368546,-0.0000860441,0.0002483406<br>O,-5.4146510838,-1.1199566886,0.3015247806<br>O,-5.4147145632,1.1196983105,-0.3013341827<br>B,4.8497597195,-0.0000075492,0.000047096<br>F,4.0158606463,1.1527879283,0.0407253103<br>F,4.0157613083,-1.1527245107,-0.0408587133<br>F,5.6442184019,-0.0407700969,1.150600486<br>F,5.6445332393,0.040676241,-1.1502961566
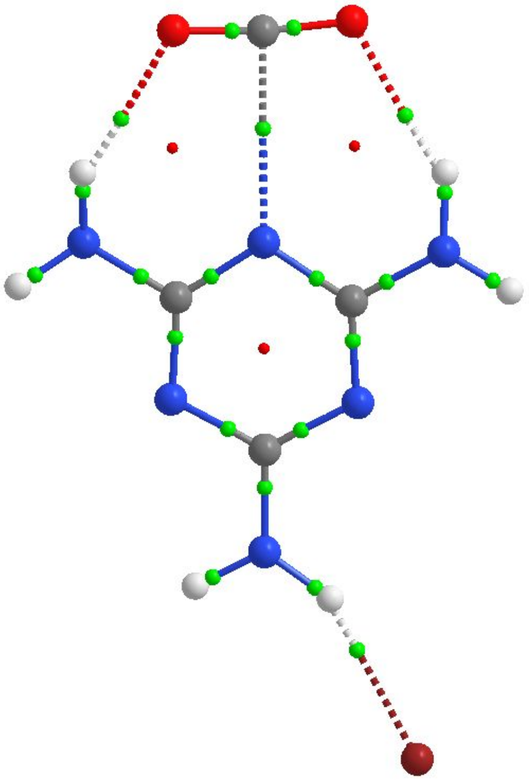	**Complex M:$Br^{(-)}$**<br>**E=-3209.72046856H; FreqIm=0**<br>C,-1.2716783156,0.5834079011,0.0037538362<br>C,0.4097590683,-0.9182841085,0.0388934716<br>C,0.8798098894,1.2753676991,-0.0519629769<br>N,0.8265121243,-2.2166656282,0.1264405646<br>H,0.1200705571,-2.9091904471,-0.0492425347<br>H,1.7714421741,-2.4179845992,-0.1461309239<br>N,-2.5667335348,0.8713157399,0.0084865953<br>H,-2.8353932306,1.8370005566,-0.0348904226<br>H,-3.3264710985,0.1786873101,0.0229323508<br>N,1.7961982144,2.2907764332,-0.1482047221<br>H,2.7369594811,2.075971701,0.1310914891<br>H,1.4433777558,3.2062425297,0.0678639494<br>N,1.370496286,0.0246576101,-0.0120203591<br>N,-0.3978052323,1.621826335,-0.036819639<br>N,-0.8976550279,-0.7118706944,0.0402371046<br>C,4.0628345895,-0.5517396289,0.0059246386<br>O,4.3612379264,0.5257045824,0.3223091949<br>O,3.8981615325,-1.6572605241,-0.3095560575<br>Br,-5.5914431593,-0.4601407678,-0.000893559

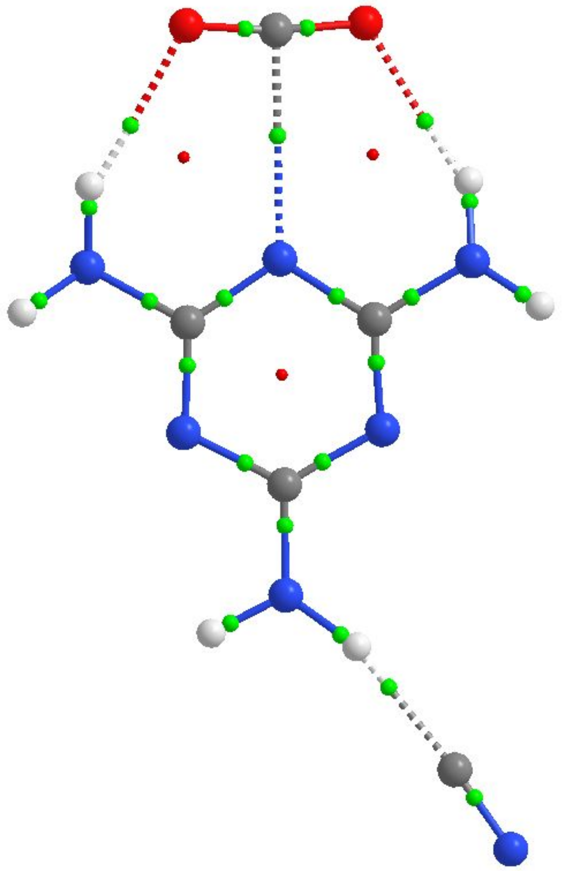	**Complex M:CN(-)**<br>**E=-728.303252747H; FreqIm=0**<br>C,1.4315905405,-0.6666799412,-0.0019523172<br>C,-0.2004008883,0.8917904874,0.038092664<br>C,-0.7463935113,-1.2833017463,-0.057553117<br>N,-0.5729331181,2.2048492965,0.130630193<br>H,0.1586078991,2.8702039795,-0.0484561368<br>H,-1.5072340578,2.4389382407,-0.1527629909<br>N,2.7143718231,-0.9978723896,0.0004294069<br>H,2.9221600971,-1.9797803402,-0.0428855892<br>H,3.5302114744,-0.3322148453,0.0155483329<br>N,-1.6992238399,-2.2671297107,-0.156815951<br>H,-2.6282913541,-2.0200813032,0.1355247402<br>H,-1.3761537848,-3.192359015,0.0650520836<br>N,-1.1945935882,-0.0166848304,-0.014733593<br>N,0.5171776743,-1.6737627618,-0.043492211<br>N,1.0978971313,0.6414664257,0.0377364093<br>C,-3.8555068799,0.6493001264,0.0142283327<br>O,-4.1906490676,-0.4131537813,0.3447453275<br>O,-3.6580147656,1.7455516944,-0.3147147154<br>C,5.2601884539,0.4266901827,0.0116869672<br>N,6.354354762,0.8317442317,0.0014741657
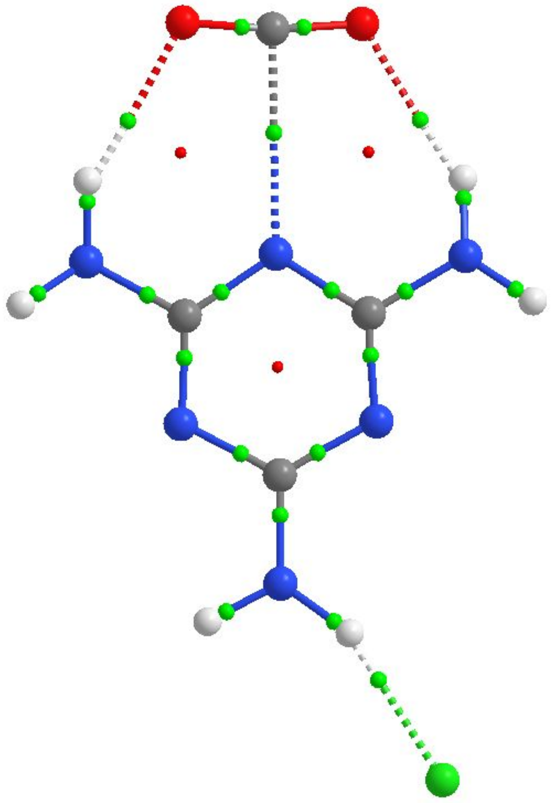	**Complex M:Cl(-)**<br>**E=-1095.71633747H; FreqIm=0**<br>C,-1.2847742066,0.5899774847,0.0018166015<br>C,0.3948987391,-0.9155981051,0.0402415461<br>C,0.8711150147,1.2759585364,-0.0552960059<br>N,0.8090457702,-2.216174035,0.131778958<br>H,0.0990195846,-2.9041735371,-0.0482267624<br>H,1.7502747786,-2.419481045,-0.1522988662<br>N,-2.5765640587,0.8823706572,0.0049064092<br>H,-2.8291415449,1.8526940321,-0.0389322737<br>H,-3.3562055619,0.1973837228,0.0212067482<br>N,1.7919572935,2.2895089613,-0.1545463525<br>H,2.7285954876,2.0717735069,0.1366556736<br>H,1.4397764529,3.2040569841,0.0672099583<br>N,1.3595279564,0.0240996203,-0.012934072<br>N,-0.4042446524,1.62601035,-0.0405230717<br>N,-0.910828925,-0.7066981015,0.0409847182<br>C,4.0409708618,-0.5577831901,0.0082114958<br>O,4.3433529442,0.5137090187,0.3411874239<br>O,3.8771783103,-1.6586797265,-0.3239317172<br>Cl,-5.3542742443,-0.5011331342,0.0107015888

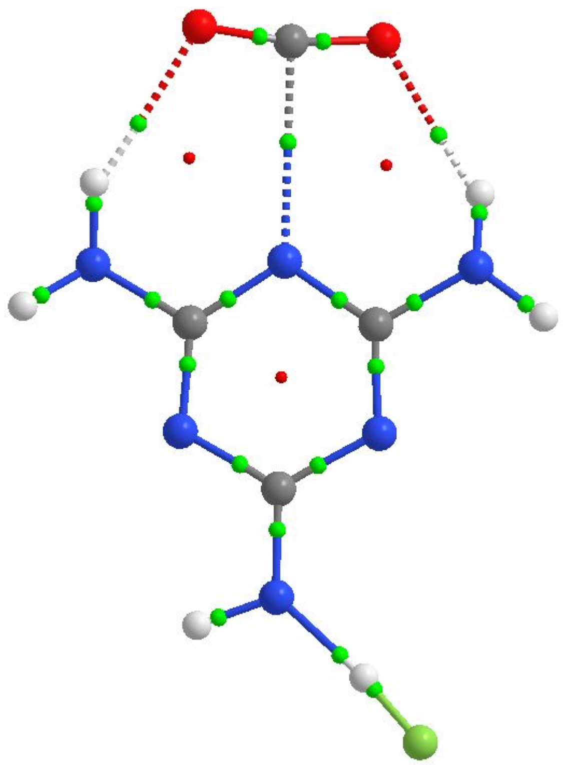	**Complex M:F$^{(-)}$**<br>**E=-735.333044438H; FreqIm=0**<br>C,-1.3141337254,0.6116419096,-0.0113663541<br>C,0.3874949548,-0.9035484457,0.0647307272<br>C,0.8742451053,1.2807588259,-0.0790778668<br>N,0.8113498941,-2.2146097096,0.194444351<br>H,0.0920146431,-2.8839661781,-0.02271634<br>H,1.7249661067,-2.4107715508,-0.1769353585<br>N,-2.5926126534,0.8825382779,-0.0180611444<br>H,-2.727195534,1.8842995647,-0.0768879342<br>H,-3.7603441466,0.0363123105,0.0244590923<br>N,1.8138789518,2.2927549043,-0.2048976428<br>H,2.7217315998,2.0769209707,0.1709517238<br>H,1.4486959086,3.1997073389,0.0329909944<br>N,1.3659894372,0.0269452677,-0.0082911601<br>N,-0.3885019694,1.6384776745,-0.0720426875<br>N,-0.9076557028,-0.6986649315,0.0560931469<br>F,-4.6638572161,-0.4786594532,0.0476942074<br>C,3.9517833672,-0.5448680354,0.0122386882<br>O,4.273532682,0.4753586805,0.4698526432<br>O,3.820676297,-1.606701421,-0.443968086

<u>Table S3:</u> Molecular graphs, energies (in Hartree) and Cartesian coordinates (in Å) of the transition states in vacuum. Level of theory: B3LYP-D3(BJ)/aug-cc-pvtz

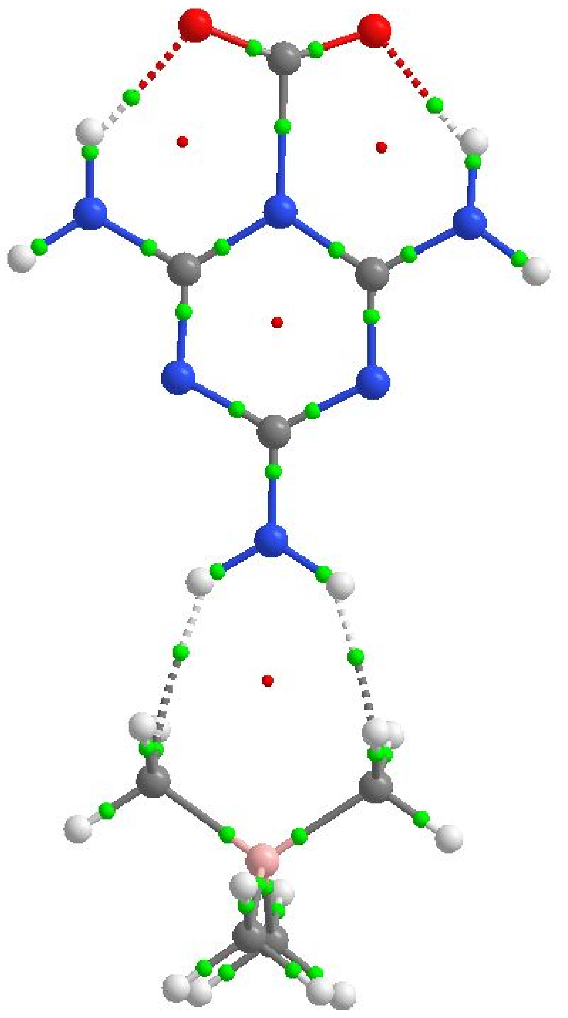	**TS M:B(CH$_3$)$_4$$^{(-)}$**<br>**E=-820.044144046H; FreqIm=1**<br>C,0.0000000016,0.0000000001,0.0275128727<br>C,-0.0447872917,1.1510202219,-1.9224697106<br>C,0.044787299,-1.1510202213,-1.9224697107<br>N,-0.1308096699,2.3254495514,-2.5759926932<br>H,-0.0353807097,3.1458447688,-2.0059336951<br>H,0.0863157392,2.3419635338,-3.5605552332<br>N,0.0000000002,0.,1.3522949367<br>H,0.0313672546,-0.8587319192,1.879361095<br>H,-0.0313672552,0.8587319191,1.8793610951<br>N,0.1308096786,-2.3254495507,-2.5759926933<br>H,-0.0863157284,-2.3419635329,-3.560555233<br>H,0.0353807172,-3.1458447682,-2.0059336956<br>N,0.0000000045,0.0000000004,-2.6582594509<br>N,0.0376316263,-1.1915264667,-0.6027445048<br>N,-0.0376316217,1.191526467,-0.6027445047<br>C,0.0000000064,0.0000000005,-4.5202463827<br>O,-0.4029176244,-1.0649149855,-4.894614199<br>O,0.402917638,1.0649149866,-4.8946141982<br>B,-0.0000000039,-0.0000000003,5.2581905184<br>C,0.0431248306,-1.3624283625,4.3194467489<br>H,0.9360063989,-1.4038165003,3.6771605457<br>H,0.0717673134,-2.2725059611,4.9320514157<br>H,-0.8450481561,-1.4599590956,3.676760948<br>C,-0.0431248364,1.3624283621,4.319446749<br>H,0.8450481516,1.4599590952,3.6767609501<br>H,-0.9360064034,1.4038164999,3.6771605439<br>H,-0.0717673205,2.2725059606,4.9320514159<br>C,-1.3491173745,-0.0427113586,6.2031935995<br>H,-1.4229522862,0.836614884,6.8579341685<br>H,-2.2743778467,-0.0720126969,5.6103148245<br>H,-1.3671717657,-0.9249180331,6.8579753426<br>C,1.3491173647,0.0427113578,6.2031936023<br>H,1.3671717545,0.9249180322,6.8579753456<br>H,1.422952275,-0.8366148849,6.8579341714<br>H,2.2743778381,0.0720126963,5.6103148294

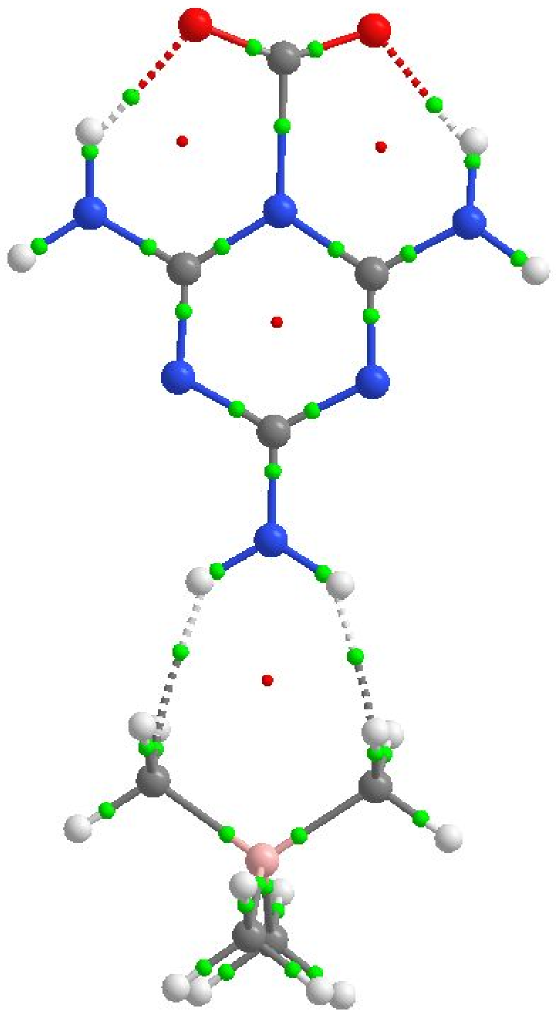	**TS M:$BF_4^{(-)}$**<br>**E=-1060.11903531H; FreqIm=1**<br>C,0.0085340149,-0.0000652935,0.0000181042<br>C,-1.9451193745,1.1482609895,0.0586245787<br>C,-1.945211821,-1.1482333229,-0.0586156878<br>N,-2.6031964781,2.3229057883,0.1631538022<br>H,-2.0334694008,3.1427773365,0.0599189122<br>H,-3.5823832571,2.3418067399,-0.0746041747<br>N,1.3286146303,-0.0001190733,0.0000268837<br>H,1.8734995705,-0.8491039325,-0.0405347146<br>H,1.873573238,0.8488195003,0.0405970449<br>N,-2.6033832429,-2.322824738,-0.1631531688<br>H,-3.5825756743,-2.3416448193,0.0745886982<br>H,-2.0337250775,-3.1427431982,-0.059910784<br>N,-2.6816583221,0.0000437204,-0.0000010364<br>N,-0.6273181859,-1.1919738923,-0.050699926<br>N,-0.6272220915,1.1918945785,0.0507275746<br>C,-4.5716459102,0.0001235078,-0.00002092<br>O,-4.9351160091,-1.060920052,0.4168678586<br>O,-4.9350162339,1.0611979659,-0.4169180636<br>B,4.7017173223,-0.0001753773,0.0000569524<br>F,3.8641321977,1.1526158691,0.0503348754<br>F,3.8640978338,-1.1529388911,-0.050239112<br>F,5.4919932975,-0.0503418771,1.1501579492<br>F,5.4920199738,0.0499684714,-1.1500266455
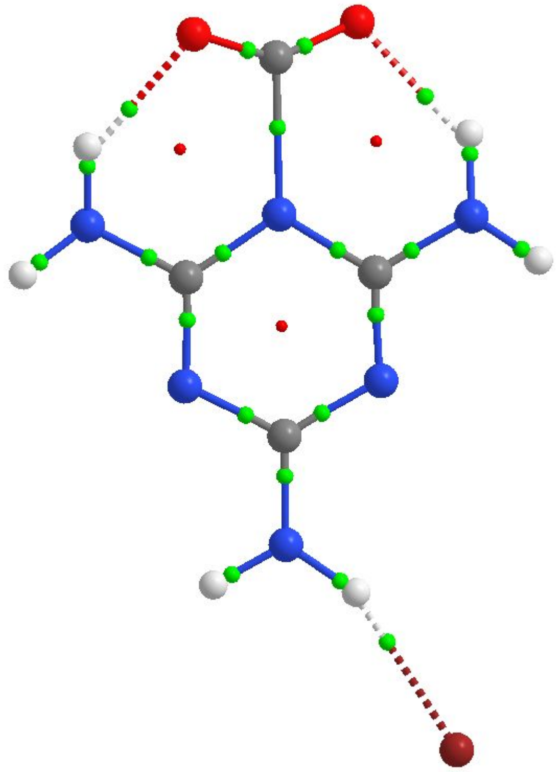	**TS M:$Br^{(-)}$**<br>**E=-3209.71418414H; FreqIm=1**<br>C,-0.2949862223,0.6744227667,-0.0197457226<br>C,1.276594901,-0.9541978267,0.0407211712<br>C,1.8997383319,1.2541682057,-0.0655624077<br>N,1.5894180294,-2.263757234,0.1406698578<br>H,0.8155293085,-2.895487373,0.0389532721<br>H,2.5263294772,-2.5519093444,-0.0898562772<br>N,-1.5643090683,1.0413898529,-0.0260645554<br>H,-1.7700396995,2.0229205958,-0.0707706643<br>H,-2.371589931,0.3950902452,-0.0000415874<br>N,2.8561259466,2.2079312874,-0.1664217239<br>H,3.7962374333,1.9576797944,0.0974203109<br>H,2.5300404551,3.1485881747,-0.0388519732<br>N,2.298122845,-0.0482314313,-0.0090100808<br>N,0.6437692057,1.6540593333,-0.0619337907<br>N,-0.003290939,-0.640564201,0.027544575<br>C,4.1338775628,-0.5633614143,0.0196997641<br>O,4.756706796,0.358702903,0.4580548298<br>O,4.1986820091,-1.677547263,-0.4071454386<br>Br,-4.5698594414,-0.2200670714,0.013657441

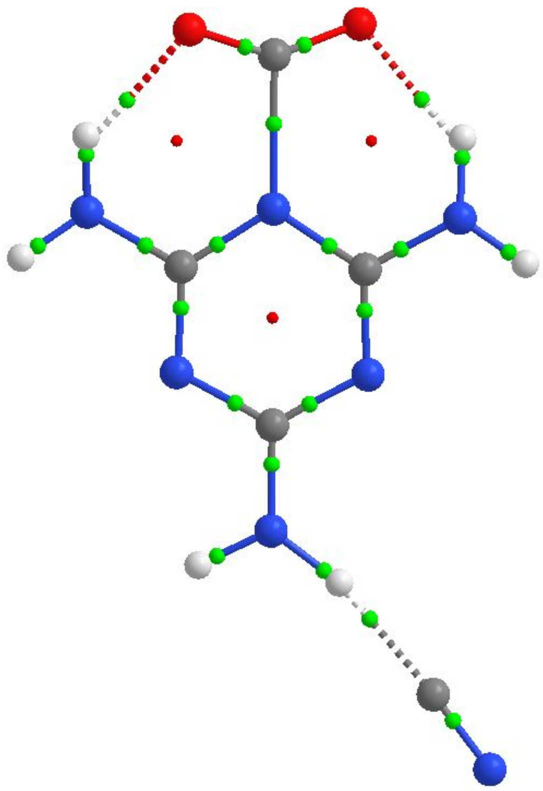

TS M:CN(-)
E=-728.297597229H; FreqIm=1
C,1.2826392796,-0.6868558774,-0.0174993119
C,-0.2825119077,0.9514474752,0.0428469094
C,-0.920051542,-1.2499477511,-0.0661323852
N,-0.5895608869,2.264482862,0.1465829676
H,0.1881153679,2.8903619468,0.0366934282
H,-1.5212045972,2.5574850896,-0.0977614204
N,2.5482781866,-1.0595669748,-0.0234197549
H,2.7194911975,-2.0491583722,-0.0679442414
H,3.3960835378,-0.4138942605,0.0030814833
N,-1.8858583524,-2.1974135216,-0.171105965
H,-2.8207202539,-1.9407893953,0.1043424318
H,-1.5661858246,-3.1388435939,-0.032449662
N,-1.3106534642,0.0538106551,-0.0092772013
N,0.3316887211,-1.6594593499,-0.0604898126
N,0.9945500808,0.6316574759,0.0301442557
C,-3.1586473773,0.585584123,0.0181001821
O,-3.7804073344,-0.3292682879,0.4699999615
O,-3.2114214847,1.6935895118,-0.4230993604
C,5.0535285875,0.3368908951,0.0272498314
N,6.1387520665,0.7629543501,0.0374086646

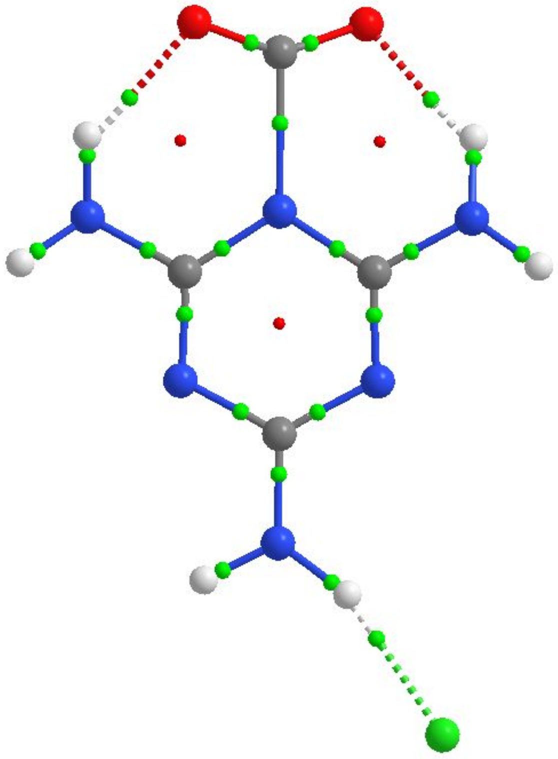

TS M:Cl(-)
E=-1095.71070884H; FreqIm=1
C,-1.1381573839,0.6150412512,-0.0169137828
C,0.4777869536,-0.971105818,0.0442888989
C,1.0433197316,1.2499421228,-0.0674449641
N,0.8267538547,-2.2733808414,0.1487417793
H,0.0692827461,-2.9238066303,0.0402515602
H,1.7673508581,-2.5365820246,-0.0951979381
N,-2.414285892,0.9496134199,-0.0224634945
H,-2.6321386763,1.9288083391,-0.0684167758
H,-3.2224850684,0.286911376,0.0050899141
N,1.9774537696,2.2282001495,-0.1736983701
H,2.9205373548,2.002619883,0.1007889145
H,1.6272564914,3.1589967319,-0.0369645623
N,1.4760516439,-0.0405502525,-0.0093131649
N,-0.2211265969,1.6184812157,-0.0617631337
N,-0.8091273209,-0.6927450184,0.0321507402
C,3.3401506472,-0.5120887655,0.0178003198
O,3.9323942766,0.4234409633,0.4669929852
O,3.4285205987,-1.6189185199,-0.4207712219
Cl,-5.1516959879,-0.4207985817,0.0250682961

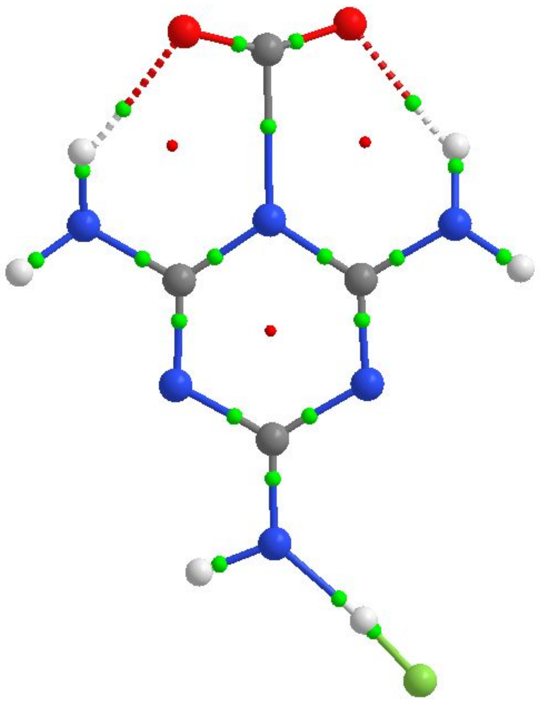	**TS M:$F^{(-)}$**<br>**E=-735.331677384H; FreqIm=1**<br>C,-1.2438633264,0.6020213318,-0.0112181895<br>C,0.4491211663,-0.9307964684,0.0609235325<br>C,0.947008198,1.2808905252,-0.0737087531<br>N,0.8606500126,-2.2361229131,0.1921834932<br>H,0.1270012985,-2.8990451545,0.0092322697<br>H,1.7772212174,-2.453303,-0.1613163101<br>N,-2.5191948208,0.8733930271,-0.0178324447<br>H,-2.6538152373,1.8756118439,-0.0700123469<br>H,-3.6998803236,0.0128376216,0.0222508172<br>N,1.8769448678,2.2888762507,-0.2008328926<br>H,2.7953301362,2.08875262,0.1594229783<br>H,1.4988704577,3.1986748385,-0.0004527156<br>N,1.4271317469,0.0131033747,-0.0070804792<br>N,-0.3158105404,1.6268901841,-0.063819193<br>N,-0.840090697,-0.7095587654,0.0482231855<br>F,-4.5925037041,-0.5011690591,0.0440564518<br>C,3.5013101111,-0.449601853,0.0084088224<br>O,3.9864073536,0.4872433107,0.5419621474<br>O,3.5502200835,-1.5047717148,-0.5211793734

Table S4: Molecular graphs, energies (in Hartree) and Cartesian coordinates (in Å) of the adducts in vacuum. Level of theory: B3LYP-D3(BJ)/aug-cc-pvtz

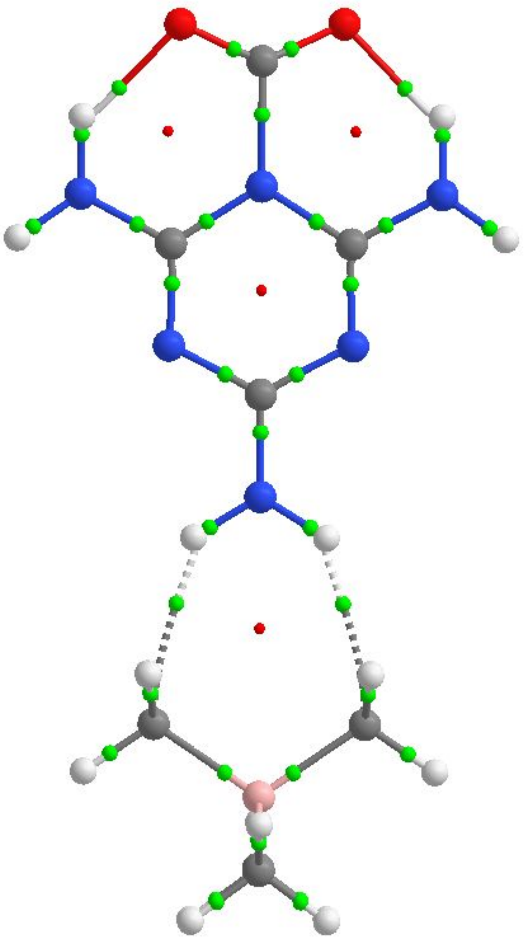	**Adduct M:$B(CH_3)_4^{(-)}$**<br>**E=-820.045106417H; FreqIm=0**<br>C,0.,0.,0.1042937212<br>C,-0.0376910722,1.1684218263,-1.8421972988<br>C,0.0376910722,-1.1684218263,-1.8421972988<br>N,-0.1102223382,2.3368791292,-2.4866105588<br>H,-0.0521329858,3.1567566311,-1.9108275048<br>H,0.0756673969,2.330658583,-3.484914044<br>N,0.,0.,1.426493954<br>H,0.0279814774,-0.8586462443,1.9552022827<br>H,-0.0279814774,0.8586462443,1.9552022827<br>N,0.1102223382,-2.3368791292,-2.4866105588<br>H,-0.0756673969,-2.330658583,-3.484914044<br>H,0.0521329858,-3.1567566311,-1.9108275048<br>N,0.,0.,-2.5864464024<br>N,0.033336316,-1.1904632796,-0.5258778695<br>N,-0.033336316,1.1904632796,-0.5258778695<br>C,0.,0.,-4.2071312467<br>O,-0.3401173813,-1.0725655421,-4.684687662<br>O,0.3401173813,1.0725655421,-4.6846876626<br>B,0.,0.,5.3027872145<br>C,0.0382559345,-1.3642644528,4.3658434113<br>H,0.931624714,-1.4117005919,3.724353496<br>H,0.0636927598,-2.2725123304,4.9809660026<br>H,-0.8508823929,-1.4615659425,3.7241689157<br>C,-0.0382559345,1.3642644528,4.3658434113<br>H,0.8508823929,1.4615659425,3.7241689157<br>H,-0.931624714,1.4117005919,3.724353496<br>H,-0.0636927598,2.2725123304,4.9809660026<br>C,-1.3493843511,-0.0378750994,6.246963169<br>H,-1.420223949,0.8416043801,6.9016576914<br>H,-2.2746747476,-0.0638487011,5.6540148225<br>H,-1.3707823116,-0.919925633,6.9016823561<br>C,1.3493843511,0.0378750994,6.246963169<br>H,1.3707823116,0.919925633,6.9016823561<br>H,1.420223949,-0.8416043801,6.9016576914<br>H,2.2746747476,0.0638487011,5.6540148225

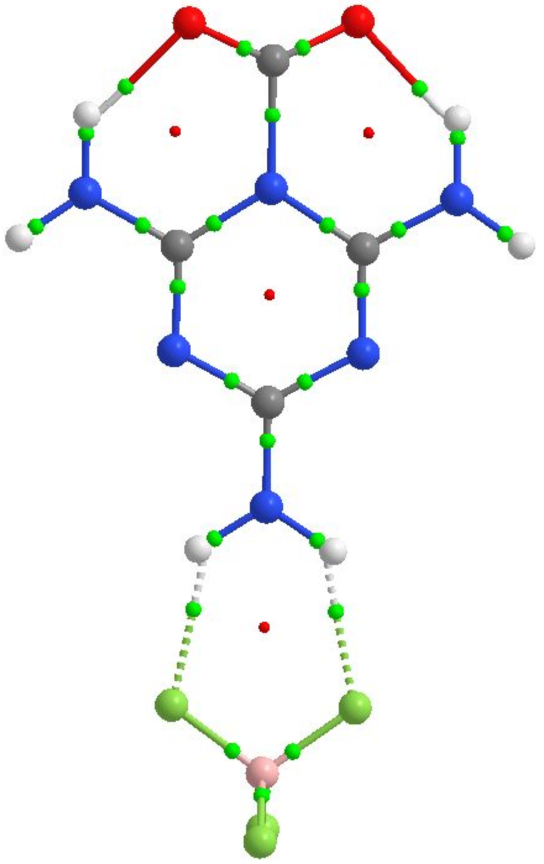	**Adduct M:$BF_4^{(-)}$**<br>**E=-1060.12052143H; FreqIm=0**<br>C,1.1712102213,0.1664694829,0.0097412885<br>C,-0.8157444536,1.2711556111,0.0184241063<br>C,-0.7388266293,-1.0660956169,-0.020357845<br>N,-1.5017013862,2.4189170627,0.0726108715<br>H,-0.9525122904,3.2553280401,-0.0051571896<br>H,-2.4949109289,2.3745754108,-0.1345070302<br>N,2.4877607968,0.2097478794,0.0170414811<br>H,3.0622985302,-0.6213674217,0.0060741095<br>H,3.0067428412,1.0763602289,0.0338606466<br>N,-1.3474745724,-2.2562978705,-0.081625907<br>H,-2.343696884,-2.2772373987,0.1143887154<br>H,-0.7455495475,-3.0548871917,0.0024509509<br>N,-1.522457471,0.0780672442,-0.005102238<br>N,0.5752490832,-1.0453355202,-0.0077175372<br>N,0.4969191177,1.3365490381,0.0202925782<br>C,-3.1310705292,0.0252309805,-0.0140479778<br>O,-3.580664556,-1.0493755138,0.3616361316<br>O,-3.6459999094,1.0681476831,-0.3951323244<br>B,5.840153031,0.3199499802,0.0356303875<br>F,4.9629600796,1.445765995,0.0456760399<br>F,5.0392248901,-0.8610167375,0.0150822469<br>F,6.6209899224,0.3305224525,1.1917016669<br>F,6.6344451942,0.3613618915,-1.1104906401
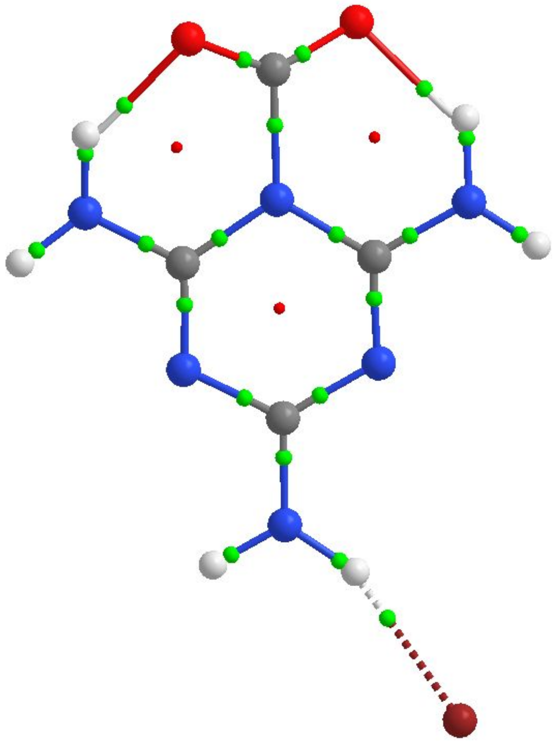	**Adduct M:$Br^{(-)}$**<br>**E=-3209.71607181H; FreqIm=0**<br>C,1.0839880784,-0.6192675131,-0.0135467599<br>C,-0.5015865757,1.0059685286,0.0261631321<br>C,-1.1034647917,-1.2533723201,-0.045796310<br>N,-0.8249853963,2.3015658283,0.1002475457<br>H,-0.054036027,2.9416694335,0.0359269625<br>H,-1.7881282378,2.5535877726,-0.0954521535<br>N,2.3557010444,-0.9663990161,-0.016264182<br>H,2.5748725126,-1.9459800234,-0.045979418<br>H,3.1566318549,-0.3055739448,0.0054281038<br>N,-2.0334447694,-2.216250764,-0.1256482804<br>H,-2.9870546811,-1.9482822707,0.1019566694<br>H,-1.6882980838,-3.1544094657,-0.038579819<br>N,-1.5243785625,0.066536534,-0.0054381442<br>N,0.1599316118,-1.6118285719,-0.0431438329<br>N,0.7728192731,0.6902912015,0.0172357641<br>C,-3.0730836048,0.4767120787,0.0136963671<br>O,-3.8098383302,-0.4252564238,0.3960347214<br>O,-3.2753226264,1.6261859426,-0.3557718938<br>Br,5.2871953114,0.3995489938,0.0285935288

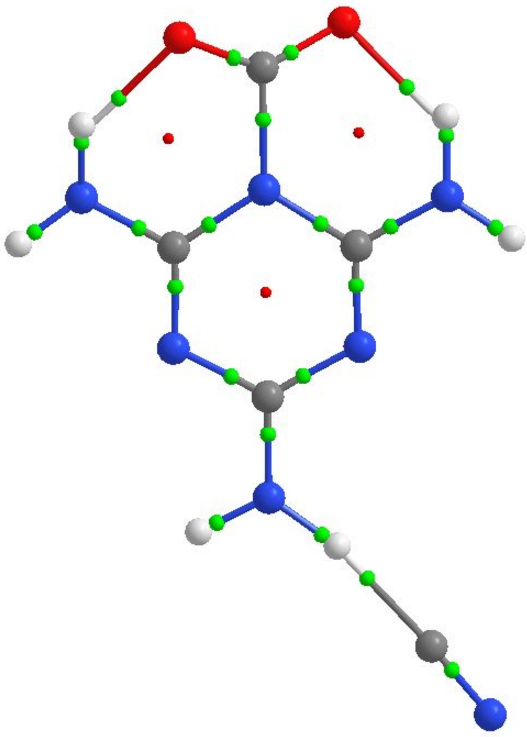	**Adduct M:CN$^{(-)}$**<br>**E=-728.299903839H; FreqIm=0**<br>C,1.1153754634,-0.6855679789,-0.0171767744<br>C,-0.4340239369,0.9787090821,0.0279329257<br>C,-1.0917177052,-1.265035931,-0.0485984185<br>N,-0.7282849007,2.2825076904,0.1084815184<br>H,0.0578213302,2.9032026439,0.0377067886<br>H,-1.683209259,2.5562611763,-0.0979341934<br>N,2.3767365173,-1.0598917566,-0.0218091169<br>H,2.54306035,-2.0513526551,-0.0535876431<br>H,3.2342982852,-0.4095265809,0.0000879017<br>N,-2.0476493549,-2.2041782624,-0.132339432<br>H,-2.9910642555,-1.9121058615,0.1083434922<br>H,-1.7251868044,-3.1498743426,-0.039918212<br>N,-1.4808219411,0.0649046637,-0.0049823005<br>N,0.1609825099,-1.6541197003,-0.0474748555<br>N,0.8307752134,0.633118745,0.0165826002<br>C,-3.0126621685,0.5108497723,0.0160043276<br>O,-3.7718215866,-0.3674541003,0.4127369715<br>O,-3.19173854,1.660831045,-0.3665451447<br>C,4.8330251688,0.3602866318,0.0241893203<br>N,5.9053516146,0.8161617194,0.0372932452
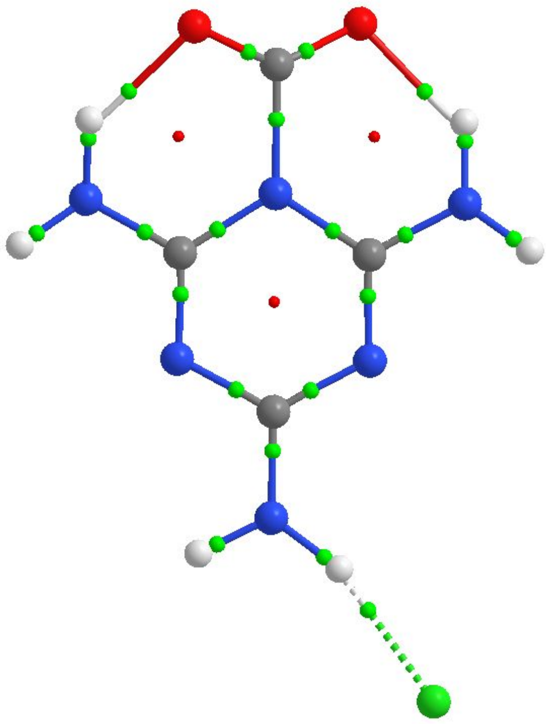	**Adduct M:Cl$^{(-)}$**<br>**E=-1095.71299706H; FreqIm=0**<br>C,1.0974766387,-0.6221857371,-0.0136802823<br>C,-0.4879253961,1.0053213038,0.0281266493<br>C,-1.0935427497,-1.2530970414,-0.048033825<br>N,-0.811348065,2.3019541507,0.1062877492<br>H,-0.0391400876,2.9401696178,0.0380573007<br>H,-1.7722628517,2.5543417754,-0.099231319<br>N,2.3662448089,-0.9713438123,-0.0164414574<br>H,2.5707600073,-1.9544313515,-0.0477225127<br>H,3.1867484746,-0.3148554958,0.0061011569<br>N,-2.0269518903,-2.2142071644,-0.132139030<br>H,-2.9776339065,-1.9445437226,0.1055306577<br>H,-1.6828358841,-3.1523320098,-0.040378114<br>N,-1.5131743772,0.0676246873,-0.0055608295<br>N,0.1679750555,-1.6131699469,-0.045131926<br>N,0.7847867094,0.6886767871,0.0190013377<br>C,-3.0557993304,0.4785839435,0.0138540327<br>O,-3.7950214884,-0.4177618878,0.4073589647<br>O,-3.2601259768,1.6250036993,-0.3665284936<br>Cl,5.0692883094,0.411698205,0.0301919422

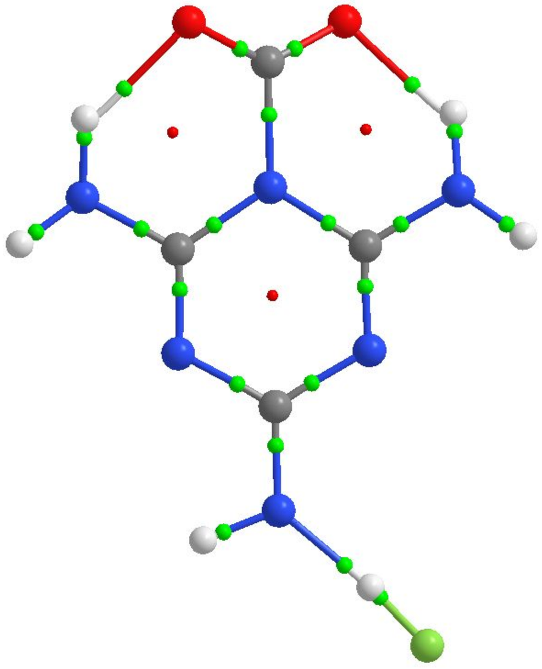	**Adduct M:$F^{(-)}$**<br>**E=-735.338250077H; FreqIm=0**<br>C,-1.2927769991,0.6191153851,-0.010320221<br>C,0.3823252793,-0.9502537048,0.0418051813<br>C,0.8996007427,1.3317242786,-0.0520137434<br>N,0.7800509601,-2.2380327393,0.155302119<br>H,0.0378394846,-2.9029983645,0.0300506948<br>H,1.7306750697,-2.4343307062,-0.141906307<br>N,-2.5630894011,0.8893577501,-0.0162283164<br>H,-2.6981933578,1.8925949938,-0.0513813365<br>H,-3.7649699011,0.0020096475,0.0112612606<br>N,1.8121635038,2.3262283912,-0.1621942889<br>H,2.7519462886,2.0896568542,0.1442813271<br>H,1.4298574808,3.2442510199,-0.0236274327<br>N,1.3765536878,0.0262800734,-0.0043760706<br>N,-0.36414475,1.6415799954,-0.0428073932<br>N,-0.8916616156,-0.6921950485,0.0279591968<br>F,-4.6395426989,-0.5131341877,0.0258429832<br>C,2.8998026702,-0.3158292981,0.0014230124<br>O,3.6134765954,0.5693725199,0.4764009174<br>O,3.1701218106,-1.42075323,-0.4691881229
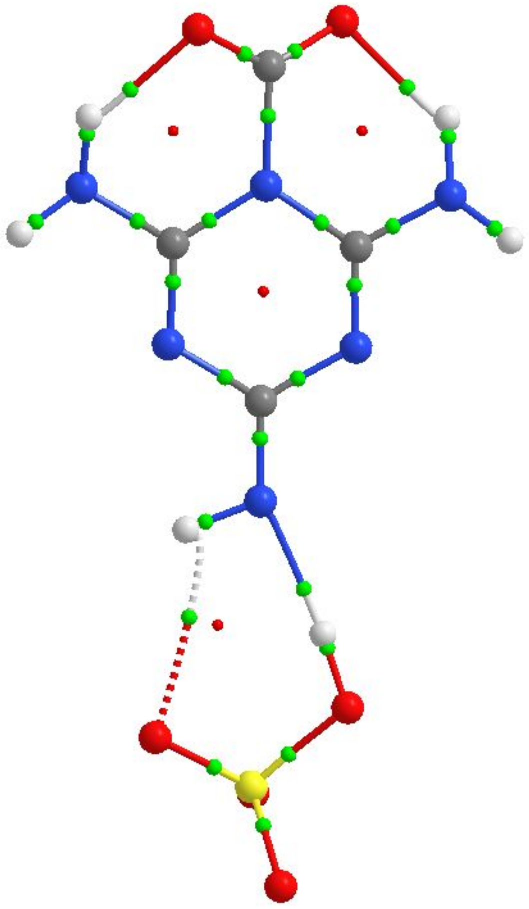	**Adduct M:$SO_4^{(-)}$**<br>**E=-1145.96017397H; FreqIm=0**<br>C,0.6112040659,-0.3057424629,-0.3133392713<br>C,2.7288939985,-0.9762186722,0.1928536114<br>C,2.2970584473,1.1974083807,-0.0517119611<br>N,3.630516429,-1.9942838556,0.5009363321<br>H,3.3274261725,-2.8924335738,0.1594741051<br>H,4.584327232,-1.7562465694,0.2824173386<br>N,-0.6342422418,-0.58079973,-0.5613989562<br>H,-1.4804781181,0.0710890358,-0.7797776003<br>H,-0.976606117,-1.5366406381,-0.566582053<br>N,2.7511464346,2.5130839446,-0.0878314845<br>H,3.5607680542,2.6723789717,0.4889468041<br>H,2.0051952421,3.1810416175,0.0244589537<br>N,3.2366949828,0.2697662927,0.2187258115<br>N,1.0259429103,1.0040815169,-0.3059967493<br>N,1.4938535973,-1.3356406039,-0.061924344<br>S,-3.8359853749,-0.7443801322,-1.2053609555<br>O,-2.9905809525,-1.9763285837,-0.954762546<br>O,-4.9071686169,-0.625333459,-0.1669655337<br>O,-2.9042440517,0.4824486451,-1.0972969801<br>O,-4.4200788236,-0.7826468942,-2.582716231

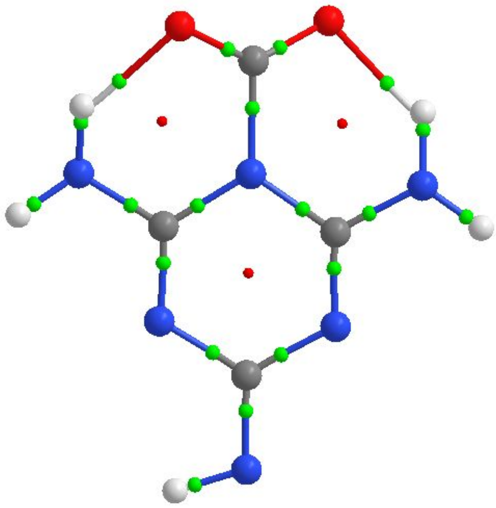

Adduct M$^{(-)}$

E=-634.582900952H; FreqIm=0

C,1.1690063393,-0.5870462395,-0.0119704462
C,-0.4867928916,1.0299259059,0.0655585663
C,-1.0610110422,-1.2483574321,-0.0675257059
N,-0.8786064373,2.3316860673,0.2242105958
H,-0.1205627819,2.9733387191,0.0214050592
H,-1.8233180191,2.5113670302,-0.1391210887
N,2.4340203343,-0.8676007961,-0.0228836376
H,2.4818237048,-1.8956595643,-0.0656274647
N,-2.0166008788,-2.2162567287,-0.2146477945
H,-2.9343935968,-1.9257537203,0.1489229729
H,-1.6496246075,-3.1379362238,-0.0069695564
N,-1.5151945319,0.0749929589,-0.0009679651
N,0.1943583869,-1.5954911527,-0.0497428548
N,0.7837064576,0.7530370312,0.0392383186
C,-3.0152407676,0.4490633244,0.0011489815
O,-3.7718222932,-0.4344511483,0.4365969068
O,-3.2700663451,1.5835099888,-0.432800877

Figure S1: Relative energy vs. N-C distance in the approach scan of the melamine:CO_2 complex.

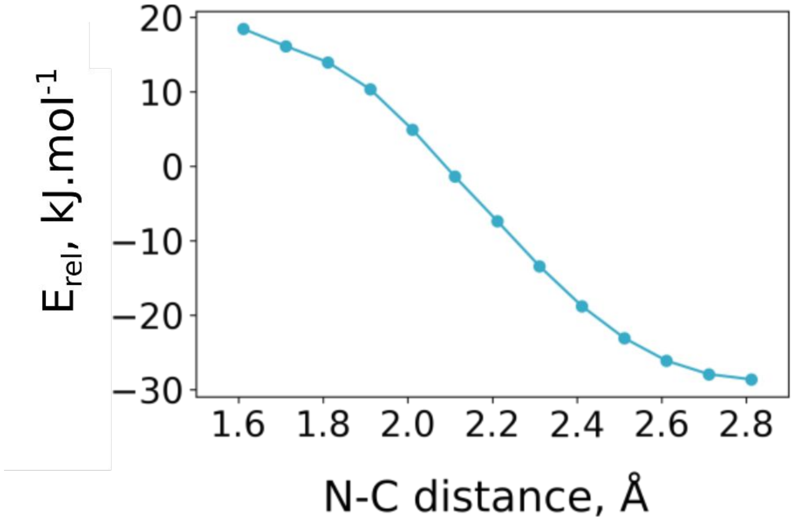

Table S5: Properties of the ortho and para complexes for the different system studied at B3LYP-D3(BJ)/aug-cc-pvtz.

Compound		E_{cplx}-($E_{monomer}$+E_{CO2}) (kJ/mol)	Distances (Å)	Angles (º)	Dipole (D)
M		-28.6	N-C : 2.83<br>O1-H1 : 2.24<br>O2-H2 : 2.24	O-C-O : 175.7<br>N-O-O-N : 0.02	0.24
M:H_2O	Ortho	-28.0	N-C : 2.84<br>O1-H1 : 2.26<br>O2-H2 : 2.24	O-C-O : 175.95<br>N-O-O-N : 0.05	1.91
	Para	-28.3	N-C : 2.84<br>O1-H1 : 2.24<br>O2-H2 : 2.24	O-C-O : 174.90<br>N-O-O-N : -4.39	1.68
M:NH_3	Ortho	-28.1	N-C : 2.84<br>O1-H1: 2.26<br>O2-H2: 2.23	O-C-O: 175.7<br>N-O-O-N : 3.46	1.61
	Para	-28.6	N-C : 2.83<br>O1-H1 : 2.24<br>O2-H2 : 2.25	O-C-O: 175.6<br>N-O-O-C: -10.67	1.30
M:NH_2CH_3	Ortho	-28.1	N-C : 2.84<br>O1-H1 : 2.26<br>O2-H2 : 2.23	O-C-O : 175.6<br>N-O-O-N : 3.72	1.52
	Para	-28.6	N-C : 2.83<br>O1-H1: 2.24<br>O2-H2 : 2.25	O-C-O: 175.6<br>N-O-O-N : -10.66	1.25
M:$NH(CH_3)_2$	Ortho	-26.4	N-C : 2.84<br>O1-H1 : 2.26<br>O2-H2 : 2.23	O-C-O: 175.46<br>N-O-O-N : -4.40	1.98
	Para	-28.6	N-C : 2.83<br>O1-H1: 2.24<br>O2-H2 : 2.25	O-C-O: 175.59<br>N-O-O-N : -10.29	0.87
M:$N(CH_3)_3$	Ortho	-28.4	N-C : 2.84<br>O1-H1 : 2.26<br>O2-H2 : 2.23	O-C-O : 175.48<br>N-O-O-N : -4.19	1.59
	Para	-28.7	N-C : 2.83<br>O1-H1 : 2.24<br>O2-H2 : 2.25	O-C-O : 175.5<br>N-O-O-N : -12.60	1.62
M:Cl-	Ortho	-28.1	N-C : 2.81<br>O1-H1 : 2.32<br>O2-H2 : 2.20	O-C-O : 174.1<br>N-O-O-N : -22.21	16.24
	Para	-31.4	N-C : 2.74<br>O1-H1: 2.27<br>O2-H2 : 2.25	O-C-O : 173.0<br>N-O-O-N : -31.04	19.67
M:Br-	Ortho	-27.8	N-C : 2.82<br>O1-H1 : 2.32<br>O2-H2 : 2.20	O-C-O : 174.3<br>N-O-O-N : -20.71	14.25
	Para	-31.1	N-C : 2.75<br>O1-H1 : 2.26	O-C-O : 173.3<br>N-O-O-N : -29.6	17.22

			O2-H2 : 2.25		
M:CN-	Ortho	-28.3	N-C : 2.81<br>O1-H1 : 2.32<br>O2-H2 : 2.20	O-C-O : 174.0<br>N-O-O-N : -21.82	18.19
	Para	-31.3	N-C : 2.74<br>O1-H1: 2.27<br>O2-H2 : 2.25	O-C-O : 173.0<br>N-O-O-N : -30.87	21.52

Table S6: REG results. In bold are indicated the most important positive terms.

**System**	**Barrier 1**		**Barrier 2**	
	**REG**	**R**	**REG**	**R**
M	Elec : -5.42	-0.99	Elec : -35.93	-0.99
	**Pauli : 10.07**	0.99	**Pauli : 76.68**	0.99
	Polar : -5.21	-0.99	Polar : -50.01	-0.99
	Def : 1.56	0.99	Def : 10.25	0.99
$M{:}OH_2$	Elec : -5.32	-0.99	Elec : -38.74	-0.99
	**Pauli : 10.12**	0.99	**Pauli : 82.89**	0.99
	Polar : -5.38	-0.98	Polar : -54.24	-0.99
	Def : 1.58	0.98	Def : 11.08	0.99
$M{:}O(CH_3)_2$	Elec : -6.47	-0.99	Elec : -68.75	-0.99
	**Pauli : 12.03**	0.99	**Pauli : 144.41**	0.99
	Polar : -6.81	-0.98	Polar : -96.33	-0.99
	Def : 2.25	0.98	Def : 21.65	0.99
$M{:}NH_3$	Elec : -6.15	-0.99	Elec : -41.38	-0.98
	**Pauli : 11.72**	0.98	**Pauli : 89.12**	0.98
	Polar : -6.50	-0.97	Polar : -58.07	-0.98
	Def : 1.93	0.97	Def : 11.33	0.97
$M{:}NH_2CH_3$	Elec : -6.29	-0.99	Elec : -47.52	-0.99
	**Pauli : 11.70**	0.99	**Pauli : 101.82**	0.99
	Polar : -6.60	-0.98	Polar : -67.47	-0.99
	Def : 2.18	0.98	Def : 14.16	0.98
$M{:}NH(CH_3)_2$	Elec : -6.23	-0.99	Elec : -46.66	-0.99
	**Pauli : 11.61**	0.99	**Pauli : 100.03**	0.99
	Polar : -6.54	-0.98	Polar : -66.28	-0.99
	Def : 2.16	0.98	Def : 13.91	0.98
$M{:}N(CH_3)_3$	Elec : -6.53	-0.99	Elec : -49.24	-0.98
	**Pauli : 12.13**	0.99	**Pauli : 105.64**	0.98
	Polar : -6.87	-0.98	Polar : -69.90	-0.98
	Def : 2.27	0.98	Def : 14.49	0.97
M:NCH	Elec : -6.71	-0.99	Elec : -77.79	-0.99
	**Pauli : 12.44**	0.99	**Pauli : 163.30**	0.99
	Polar : -7.07	-0.98	Polar : -108.89	-0.99
	Def : 2.33	0.98	Def : 24.37	0.99
$M{:}B(CH_3)_4^{(-)}$	Elec : -11.86	-0.97	Elec : 80.62	0.99
	**Pauli : 21.52**	0.96	Pauli : -166.68	-0.99
	Polar : -12.98	-0.95	**Polar : 111.51**	0.99
	Def : 4.31	0.96	Def : -24.43	-0.99
$M{:}BF_4^{(-)}$	Elec : -11.40	-0.97	Elec : 69.73	0.99
	**Pauli : 20.16**	0.96	Pauli : -142.20	-0.99
	Polar : -13.21	-0.95	**Polar : 98.14**	0.99
	Def : 5.46	0.97	Def : -24.65	-0.99
$M{:}Br^{(-)}$	Elec : -12.85	-0.97	Elec : 54.76	0.99
	**Pauli : 23.21**	0.97	Pauli : -113.01	-0.99
	Polar : -13.38	-0.95	**Polar : 74.75**	0.99
	Def : 4.02	0.95	Def : -15.49	-0.99
$M{:}CN^{(-)}$	Elec : -13.28	-0.98	Elec : 55.50	0.99
	**Pauli : 23.38**	0.98	Pauli : -113.83	-0.99

	Polar : -13.97<br>Def : 4.86	-0.97<br>0.97	**Polar : 76.02**<br>Def : -16.68	0.99<br>-0.99
M:Cl$^{(-)}$	Elec : -12.38<br>**Pauli : 22.03**<br>Polar : -12.43<br>Def : 3.78	-0.99<br>0.98<br>-0.97<br>0.97	Elec : 51.89<br>Pauli : -106.33<br>**Polar : 70.39**<br>Def : -14.93	0.99<br>-0.99<br>0.99<br>-0.99
M:F$^{(-)}$	Elec : -32.27<br>**Pauli : 53.72**<br>Polar : -32.12<br>Def : 11.65	-0.98<br>0.98<br>-0.97<br>0.97	Elec : 27.75<br>Pauli : -55.88<br>**Polar : 37.40**<br>Def : -8.26	0.98<br>-0.98<br>0.98<br>-0.99
